# Evaluation of nail-plate construct fixation for complicated distal tibial fractures: a retrospective analysis of clinical and radiographic outcomes

**DOI:** 10.1007/s00590-026-04704-2

**Published:** 2026-04-15

**Authors:** I-Jung Chen, Yi Lu, Shu-Chen Liao, Chang-Heng Liu, Po-Ju Lai, Sheng-Hsuan Lin

**Affiliations:** 1 Division of Orthopedic Traumatology, Department of Orthopedic Surgery, Chang Gung Memorial Hospital, Taoyuan, Taiwan; 2 Graduate Degree Program of Biomedical Science and Engineering, National Yang Ming Chiao Tung University, Hsinchu, Taiwan; 3 Department of Orthopedic Surgery, Chang Gung Memorial Hospital, Taoyuan, Taiwan; 4 Department of Orthopedic Surgery, New Taipei Municipal TuCheng Hospital, New Taipei City, Taiwan; 5 Institute of Statistics, National Yang Ming Chiao Tung University, Hsinchu, Taiwan; 6 Institute of Data Science and Engineering, National Yang Ming Chiao Tung University, Hsinchu, Taiwan; 7 Department of Applied Mathematics, National Dong Hwa University, Hualien, Taiwan; 8 Department of Biochemical and Molecular Medical Sciences, National Dong Hwa University, Hualien, Taiwan

**Keywords:** Distal tibial fracture, Intramedullary nail, Tibia plate, Nail plate construct

## Abstract

**Purpose:**

Distal tibial fractures are complex injuries and present significant management challenges owing to their proximity to the ankle joint, poor vascular supply, and extensive soft tissue damage. The nail-plate construct (NPC) has been proven to be a solid fixation method for multiple types of complicated open or closed fractures. However, the use of the NPC in patients with complicated distal tibial fractures has not been comprehensively reported.

**Methods:**

This case series involved eight patients who underwent NPC for distal tibial fractures. We assessed the postoperative radiographic union status and functional outcomes of patients using the Olerud–Molander Ankle Score (OMAS) and the American Orthopedic Foot and Ankle Society (AOFAS) score. A web-based questionnaire featuring the presentation of three cases treated with NPC was distributed to orthopedic trauma surgeons to gauge their perceptions of NPC following a review of the study results.

**Results:**

At the 12-month follow-up, the average OMAS and AOFAS scores were 77.63 and 82, and the average time to radiographic union and weight bearing was 7.8 and 3.4 months, respectively. Following the questionnaire, 95% of the surveyed surgeons indicated a willingness to consider NPC as a definite fixation method for future cases of complicated distal tibial fractures, demonstrating increased acceptance of this technique.

**Conclusions:**

The NPC technique has good functional and radiographic outcomes for complicated distal tibial fractures and has promising potential for broader clinical acceptance. We advocate for the application of the NPC technique in specific cases, such as combined Arbeitsgemeinschaft für Osteosynthesefragen/Orthopedic Trauma Association 42 and 43 tibial fractures, distal tibial fracture nonunion, and open fractures with bone defects.

**Supplementary Information:**

The online version contains supplementary material available at 10.1007/s00590-026-04704-2.

## Introduction

Distal tibial fractures are complex injuries predominantly caused by high-energy impacts, such as motor vehicle accidents, mainly affecting young adults and occurring more frequently in male individuals [1, 2]. These fractures represent a significant challenge in orthopedic practice owing to their proximity to the ankle, the inherent poor vascular supply of the distal tibia, and the common presence of extensive soft tissue damage [3, 4]. The pathophysiology of these fractures involves not only direct trauma but also the transmission of force along the bone axis from the ankle, often leading to comminution and displacement, making them difficult to manage and often resulting in long-term disability [5].

Non-operative treatments, such as casting or bracing, are typically reserved for less severe fractures where the risk of surgical complications is deemed too high or the patient’s overall health precludes invasive procedures [6]. Surgical options are necessary in more severe cases to ensure proper alignment, stability, and fracture healing while minimizing additional soft tissue damage. Techniques include minimally invasive plate osteosynthesis (MIPO), which preserves soft tissue integrity and reduces the risk of infection and skin necrosis [7]. Intramedullary nailing (IMN) is used for its stability and facilitation of early mobility, although meticulous execution is necessary to avoid complications, such as malalignment or chronic knee pain [5]. External fixation is often employed in cases with severe soft tissue damage or when immediate definitive surgery is not feasible. Although this helps to manage soft tissue conditions, it has the potential to lead to higher rates of non-union and infection [8].

Intramedullary nail and plate construct (NPC) fixation is an additional surgical option for complicated distal tibial fractures. This method offers the potential for enhanced stability and alignment, particularly in complex fracture patterns where other methods might fail to provide sufficient support and alignment [9, 10]. The use of NPC addresses different aspects of the fracture, ensuring comprehensive management of both the alignment and integrity of the surrounding soft tissue, as addressed by Yoon et al. [11]. NPC has become increasingly common for treating complicated distal femoral and proximal tibial fractures, but few studies have reported its use for distal tibial fractures [5].

To the best of our knowledge, this case series represents the largest cohort study focusing on the application of NPC in the management of distal tibial fractures. In this case series, we evaluated the radiographic and functional outcomes of patients with complicated distal tibial fractures who underwent NPC at our institution. By presenting these cases, we provide surgeons with viable treatment options for similar fractures.

## Methods

### Study population

We conducted a single-center retrospective review to assess the efficacy of NPC as a surgical treatment for patients with complicated distal tibial fractures. The study period was from January 1, 2020, to May 31, 2023. The inclusion criteria comprised closed or open distal tibial fractures diagnosed based on clinical presentation, plain radiography, and three-dimensional computed tomography (3D CT). All included patients underwent internal fixation using the NPC method and completed radiographic and clinical follow-up for a minimum of 1 year. Both revision and primary surgeries were accepted for inclusion. The indications for NPC included segmental (combined Arbeitsgemeinschaft für Osteosynthesefragen/Orthopedic Trauma Association [AO/OTA] 42 and 43) fractures, nonunion of distal tibial fractures, and open fractures with bone defects. Patients were excluded if they refused to provide the required functional outcome data for the study, were aged < 18 years, or had a follow-up < 1 year.

### Surgical technique

The patient was positioned on the operating table in the supine position. Depending on the surgeon’s preference, previous external fixator pins, if present, were used as distractors. Alternatively, new pins were placed to aid in the manipulation of fractures and evaluation of the articular surface.

The fixation order was tailored to each case. Generally, the plate was placed first using the MIPO technique. This approach was chosen to maintain the length and rotational alignment of the tibia while allowing the distal portion of the tibia to move as a single unit. The surgical approach was based on the fracture pattern, with the anterolateral approach being predominantly used owing to soft tissue concerns. In some cases, a posteromedial approach was preferred.

The articular surface of the tibial plafond was elevated using a combination of wires, osteotomes, bone tamps, and/or bone graft as needed. The distal end of the plate was then positioned and provisionally secured with K-wires or a single screw. A unicortical screw was used to stabilize the proximal part of the plate. It was crucial to plan the screw trajectory carefully to avoid the nail path and to provide support for the articular surface fragments. If the screw trajectory was suboptimal, additional rafting screws or miniplates separate from the primary plate were used. Initially, the screws were made short and were later replaced with bicortical or longer screws once the nail was in position.

The nail component was placed according to the surgeon’s preference, with the aim to position it as distally as possible to maximize distal tibial fixation with the nail locking bolts. Nails with multiple distal screw options were preferred, allowing screws to be directed into different parts of the plafond and providing additional support to distal fragments. When the relative positions of the nail and plate were optimal, screws were placed through both the plate and distal interlocking holes of the nail to effectively link the constructs. However, this nail-plate linkage is not strictly necessary if the plate is securely fixed in the tibial shaft.

### Post-operative evaluation

All patients underwent identical postoperative care during their admission phase and were discharged with scheduled outpatient follow-up appointments at 2 weeks, 6 weeks, 3 months, 6 months, and 12 months after discharge, with annual follow-up conducted thereafter. Anteroposterior and lateral radiographs of the tibia and ankle were obtained at each follow-up visit to evaluate the radiographic union status. Postoperative 3D CT scans were performed to assess the reduction quality if the articular surface was severely injured. Functional evaluations were performed and documented during each clinic visit. Functional results were evaluated using the Olerud–Molander Ankle Score (OMAS) and the American Orthopedic Foot and Ankle Society scoring system (AOFAS) [12, 13]. Perioperative or postoperative complications, including neurovascular injury, loss of reduction, nonunion, malunion, hardware failure or irritation, infection, and soft tissue necrosis, were recorded.

### Web-based questionnaire

A web-based questionnaire was developed to collect opinions on the use of NPC for complicated distal tibial fractures among fellowship-trained orthopedic trauma surgeons. The questionnaire briefly described the history of the three included cases and provided images, including preoperative radiographs and 3D CT scans of the patients. Surgeons were initially asked about their preferences for treatment and implant selection. Subsequently, the surgical technique involving NPC fixation was outlined, followed by a demonstration of postoperative images and the clinical conditions. A question asking whether surgeons would choose NPC as their fixation method after reviewing this information was included in the last part of the questionnaire. The questionnaire is included in the Supplement Material.

### Statistical analysis

Statistical analysis was performed using the IBM SPSS Statistics 25 (IBM, Armonk,

New York, USA). Continuous variables are presented as means with ranges, while categorical data are expressed as absolute frequencies and percentages. Due to the small cohort size and the lack of a comparative control group, descriptive statistics were utilized to summarize the clinical outcomes and survey responses.

## Results

This case series investigated the outcomes of eight patients diagnosed with complicated distal tibial fractures treated with NPC for open reduction and internal fixation (ORIF). The cohort comprised four male and four female patients, with an average age of 43.7 (range: 29–56) years. The primary mechanisms of injury were high-energy impacts, such as motor vehicle accidents, crush injuries, and falls from significant heights. The average Injury Severity Score was 10.25 (range: 4–25). Five of the eight patients were diagnosed with open fractures, and according to the Gustilo–Anderson classification, three fractures were categorized as type III, and one each as type I and II. Notably, all patients presented with significant soft tissue damage, necessitating the initial use of external skeletal fixation for stabilization, followed by definitive ORIF in six patients. Regarding comorbidities, two patients had type II diabetes mellitus, one had a history of gout, and another had hypertension. The follow-up period ranged from 12 to 36 months, with an average of 24 months (Table 1).

**Table 1:** Patients’ demographic data and fracture type

Case No	Age (y)	Sex	BMI	Mechanism	ISS	Comorbidity	Gustilo classification	AO/OTA class	Initial ESF
*1	57	Male	24	Crush	9	Gouty arthritis	IIIb	42B3 + 43C2.3	+
*2	70	Male	21.4	Crush	18	T2DM, HTN	II	43A3.1	+
*3	39	Female	28.6	MVA	4	–	Closed	42B2 + 43C3.3	+
4	50	Male	24.2	MVA	9	–	I	43A3.2	+
5	40	Male	26.9	MVA	25	–	IIIa	42B3 + 43A1.2,	+
6	33	Female	24.1	MVA	4	–	Closed	42A2 + 43A1.2	–
7	22	Female	29.7	MVA	9	–	IIIb	43A3.2	+
8	39	Female	36.5	Falling accident	4	T2DM	Closed	42B2 + 43B1.2	–

Cases numbered 1–3, each marked with a star, were presented in the questionnaire, detailing their brief history and surgical management with NPC. MVA: Motor vehicle accidents, T2DM: Type II diabetes mellitus, HTN: Hypertension, NPC: Nail-plate combination, AO/OTA: Arbeitsgemeinschaft für Osteosynthesefragen/Orthopedic Trauma Association, ISS: Injury Severity Scale, BMI: body mass index, ESF: external skeletal fixation

The average time to radiographic bone union was 7.8 (range: 6–14) months. One patient with poor-controlled diabetes mellitus experienced the longest union time of 14 months. Patients without significant comorbidities or complications typically achieved bone union by the 6-month follow-up, which is consistent with the expected recovery timeline for healthy adults undergoing similar treatments. The average time to weight bearing was 3.4 (range: 2.5–6) months. Regarding functional outcomes, the average OMAS score was 77.63 (range: 68 to 85), and the average AOFAS score was 82 (range: 70 to 88). The scores indicate good functional outcomes at 12 months after surgery (Table 2).

**Table 2:** Patients’ postoperative status, including radiographic and functional outcome

Case No	Time to weight bearing (months)	Radiographic union time (months)	OMAS score	AOFAS score	Complications
*1	3	6	85	82	–
*2	6	9	68	84	–
*3	2.5	14	78	83	Nonunion
4	4	6	85	88	–
5	3	9	68	70	–
6	3	6	81	83	–
7	3	6	72	80	–
8	3	6	84	86	–

Cases numbered 1–3, each marked with a star, were presented in the questionnaire, detailing their brief history and surgical management with nail-plate combination. Functional scores were recorded at 12 months after surgery. OMAS: Olerud–Molander Ankle Score, AOFAS: American Orthopaedic Foot and Ankle Society scoring system

Complications were reported in one patient who was a heavy smoker who exhibited nonunion after 12 months of follow-up. Revision surgery was performed using autologous bone grafting, resulting in successful fracture union. No cases of neurovascular injury, loss of reduction, hardware failure, infection, or soft tissue necrosis were detected during follow-up. No significant coronal or sagittal plane malunion was observed.

**Fig. 1:**
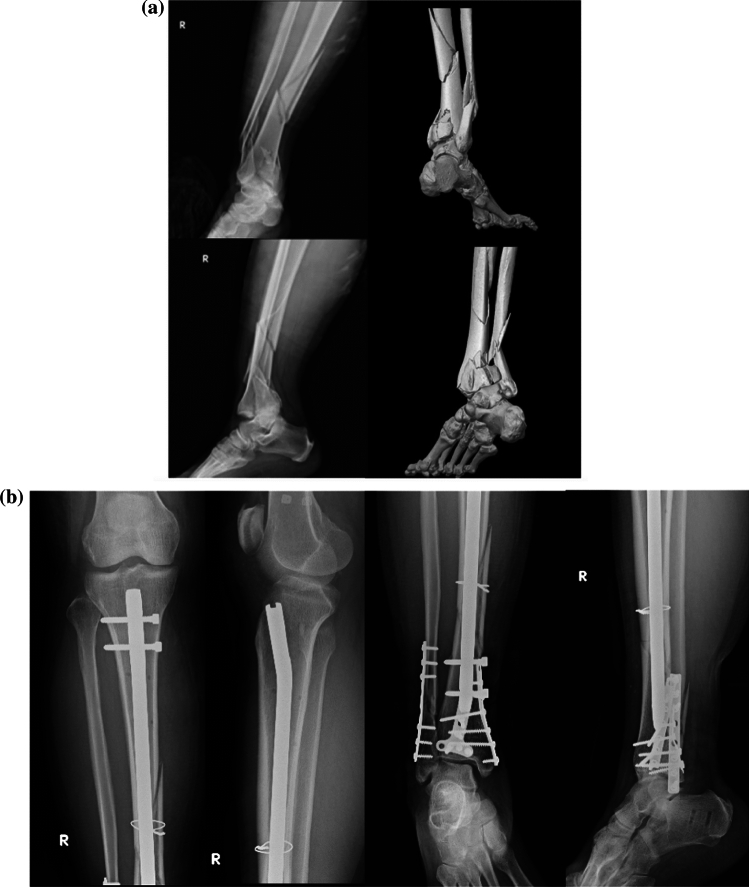

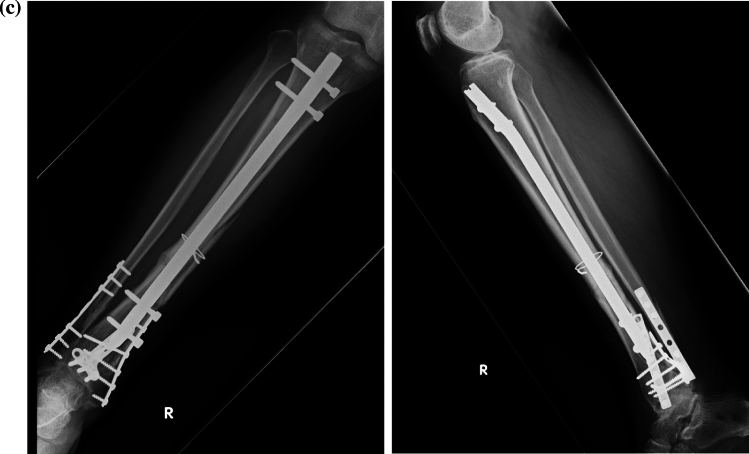
Representative case of a 39-year-old woman involved in a motorbike accident. **a** Radiographs and CT of a right tibia AO/OTA 42B2 + 43C3.3 fracture. **b** Post-operative radiographs after the fracture was operated with a nail-plate construct. **c** Radiograph 6 months after the operation showing bone union. CT: computed tomography, AO/OTA: Arbeitsgemeinschaft für Osteosynthesefragen/Orthopedic Trauma Association

**Fig. 2:**
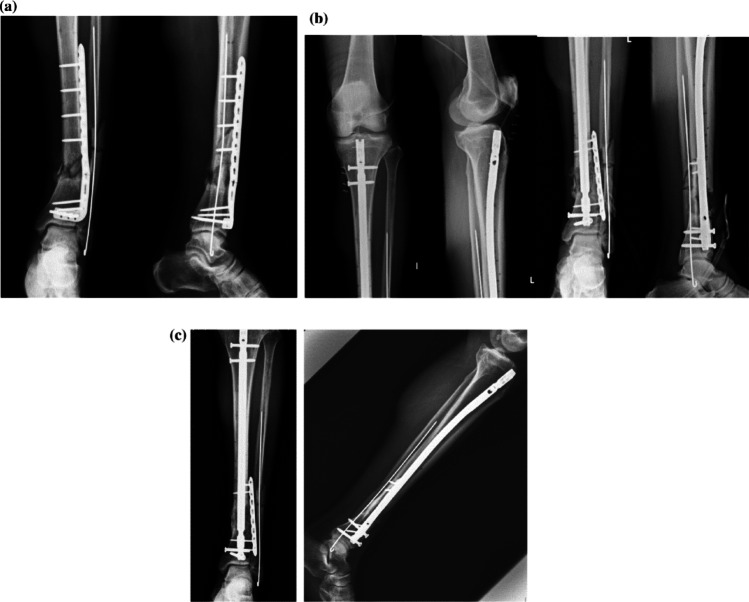
Representative case of a 70-year-old man after a pedestrian injury. **a** Four months after plate fixation for the distal tibial fracture, radiographs demonstrate fracture nonunion and implant failure. **b** Post-operative radiographs after the patient was operated through a nail-plate construct. **c** Nine months after the revision surgery, bone union is noted and the patient could walk freely without support

**Fig. 3:**
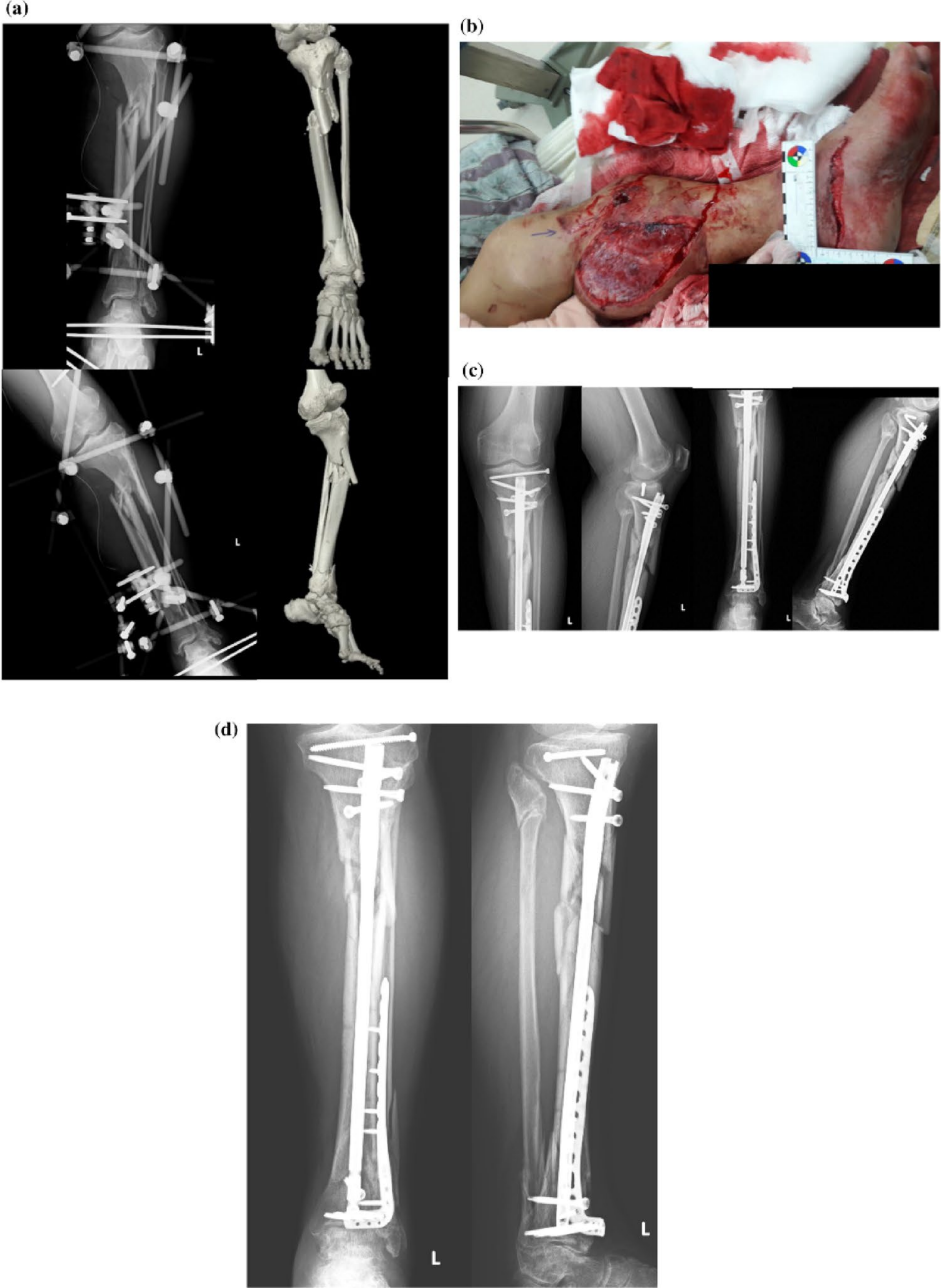
Representative case of a 57-year-old man after his left lower limb was hit by a steel rod during work. **a** Radiographs and CT of a left tibia AO/OTA 42B3 + 43C2.3 fracture. **b** Photograph showing a Gustilo open type III fracture with severe soft tissue damage. **c** Post-operative radiographs after the patient was operated through a nail-plate construct. **d** Radiograph 6 months after the operation showing bone union. CT: computed tomography, AO/OTA: Arbeitsgemeinschaft für Osteosynthesefragen/Orthopedic Trauma Association

The respondents of the questionnaire comprised a group of 20 surgeons, each with at least 5 years of attending experience, from various medical centers and regional hospitals, handling more than 15 major orthopedic trauma surgeries monthly. Specifically, in addition to Taiwanese and Chinese surgeons, two Korean and two Thai surgeons completed the questionnaire, thereby enriching the versatility of the responses. The results revealed a strong preference for ORIF using a combination of intramedullary nailing and locking compression plates (LCPs). Pre- and postoperative radiographs and 3D CT images for cases 1 (Fig. 1), 2 (Fig. 2), and 3 (Fig. 3) were presented in the questionnaire. Three cases selected for pictorial representation were chosen because they best illustrate the spectrum of complexity and the specific surgical challenges addressed by the NPC technique.

**Fig. 4:**
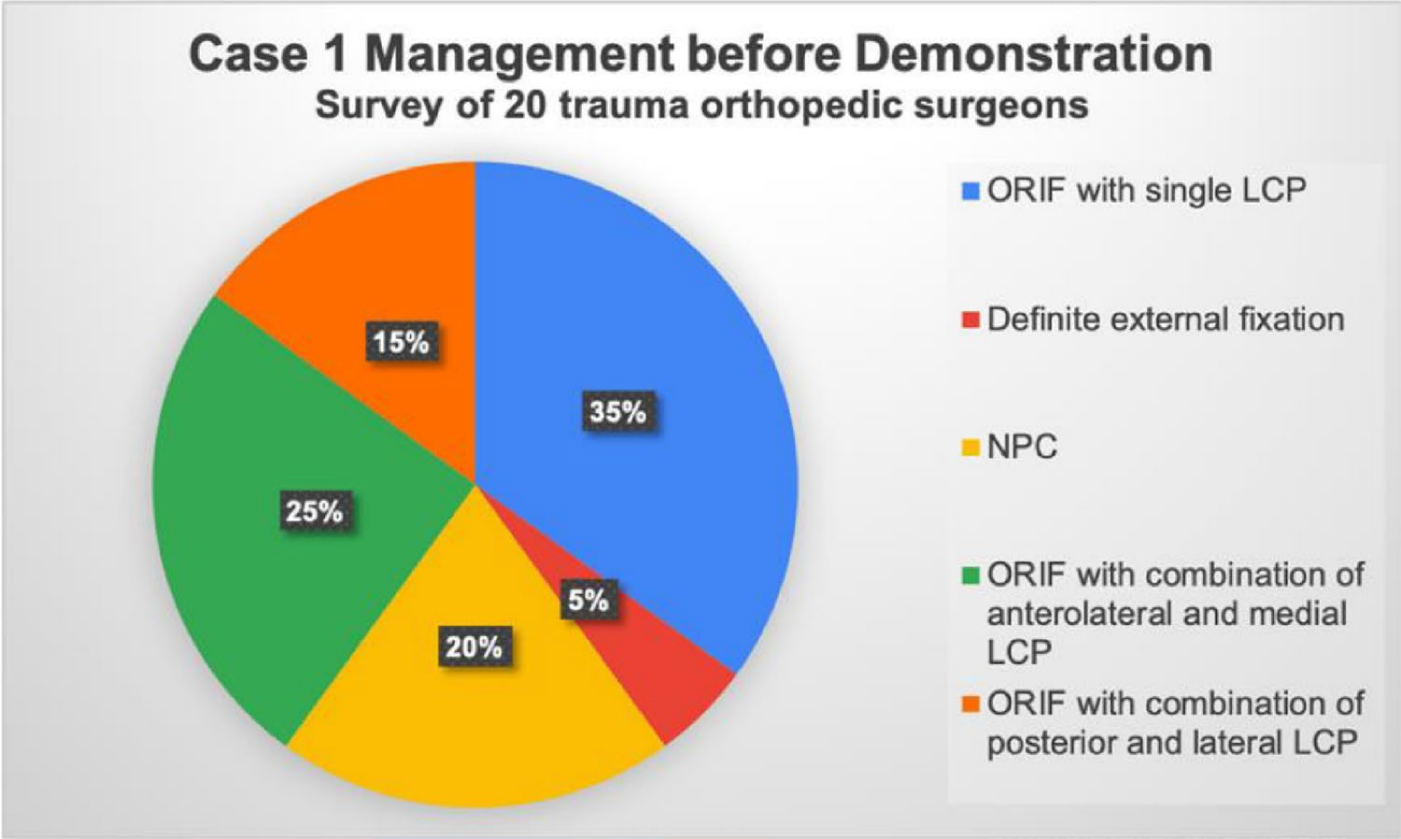
Surgeons’ responses regarding their preferred surgical management for Case 1, prior to the demonstration of NPC management. ORIF: open reduction and internal fixation, LCP: locking compression plate, NPC: nail-plate construct

**Fig. 5:**
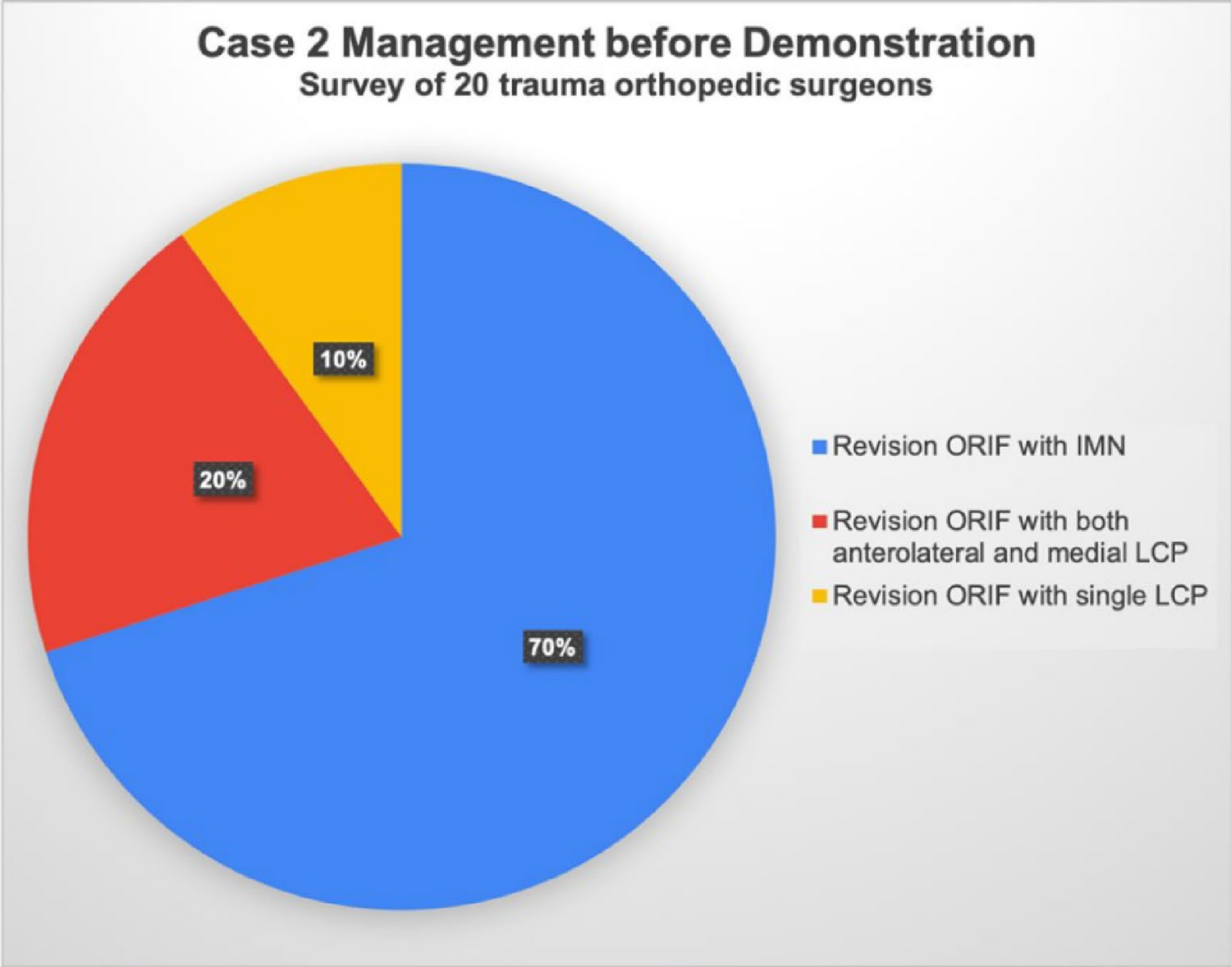
Surgeons’ responses regarding their preferred surgical management for Case 2, prior to the demonstration of NPC management. ORIF: open reduction and internal fixation, IMN: intra-medullary nails, LCP: locking compression plate, NPC: nail-plate construct

**Fig. 6:**
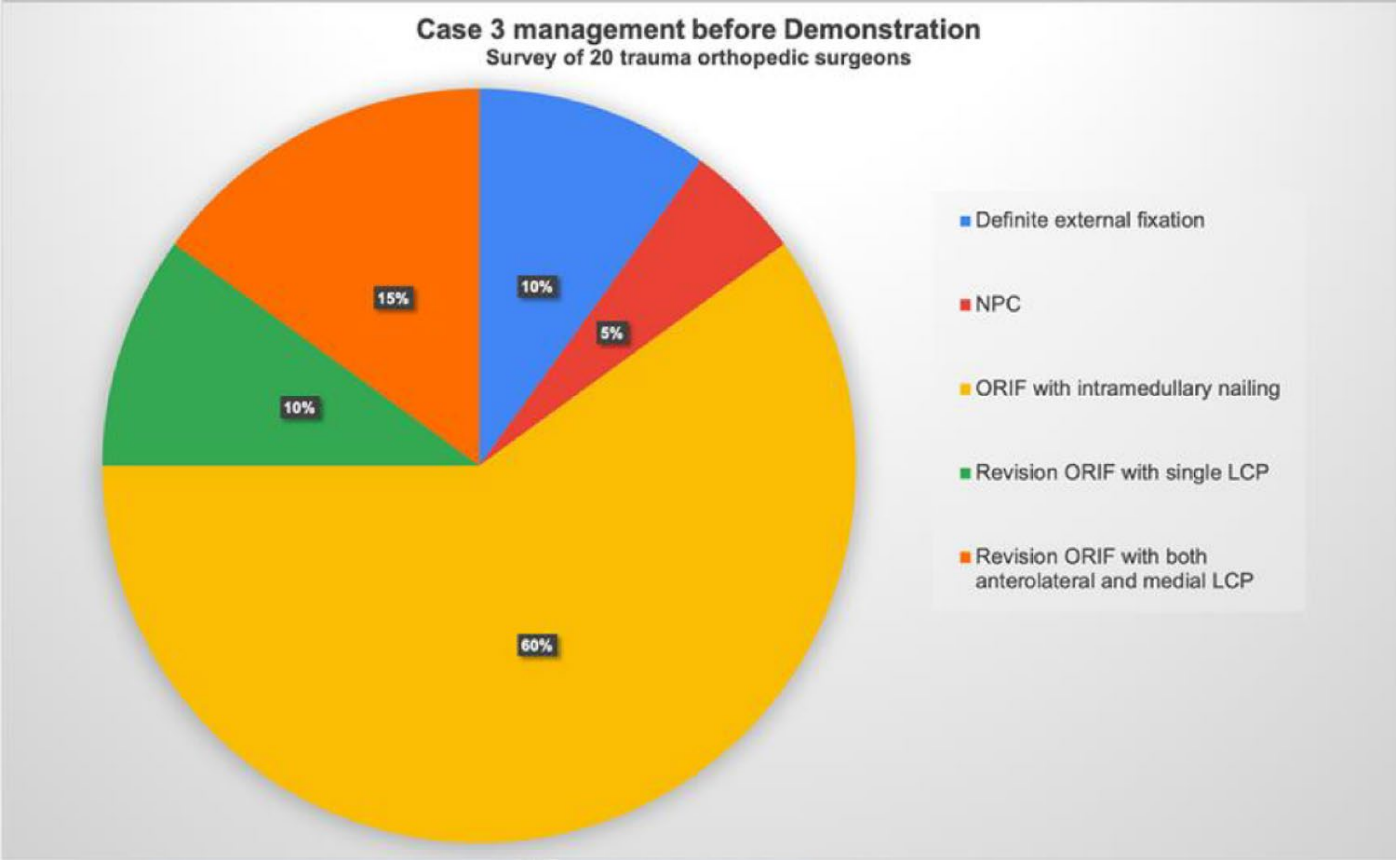
Surgeons’ responses regarding their preferred surgical management for Case 3, prior to the demonstration of NPC management. NPC: nail-plate construct, ORIF: open reduction and internal fixation, LCP: locking compression plate

**Fig. 7:**
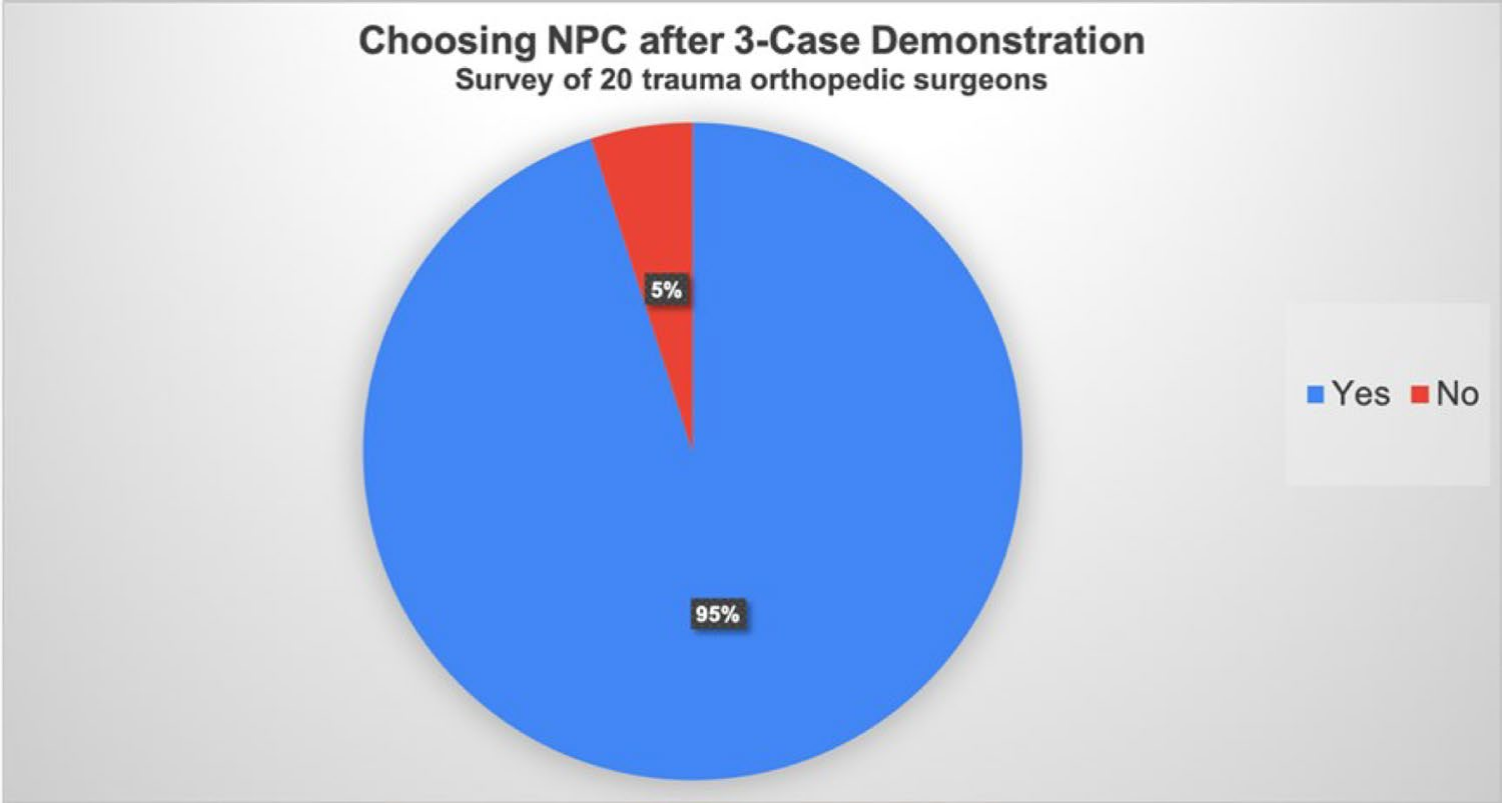
Surgeons’ responses regarding their selection of NPC as the surgical management after the presentation of the three cases utilizing NPC as the ORIF method. NPC: nail-plate construct, ORIF: open reduction and internal fixation

Specifically, for Case 1, the treatment preferences among the surgeons were as follows: Seven opted for ORIF with a single locking plate, five chose a combination of anterolateral and medial LCPs, three preferred posterior and lateral LCPs, and one opted for definite external fixation. Additionally, four surgeons recommended NPC for this patient (Fig. 4). In Case 2, intramedullary nailing was the preferred treatment, with no surgeons recommending NPC. Moreover, four surgeons chose revision ORIF with both anterolateral and medial LCPs, and two preferred a single LCP (Fig. 5). In Case 3, 12 surgeons favored intramedullary nailing, and one recommended NPC. Two surgeons opted for a single LCP, and three chose both anterolateral and medial LCPs for ORIF revision. Notably, two surgeons selected definite external fixation for this case (Fig. 6). After the demonstration of our surgical intervention with NPC and the postoperative radiograph with a paragraph specifically introducing the application of NPC to complicated distal tibial fractures, nearly every surgeon (19 of 20) responded that they would like to utilize NPC as their fixation method (Fig. 7).

## Discussion

The management of distal tibial fractures poses significant challenges because of their proximity to the ankle joint, poor vascular supply, and the often-extensive soft tissue damage associated with these injuries [14]. Traditional methods, such as non-operative treatments and surgical techniques, have been used with varying degrees of success. Recently, NPC has been proposed as a viable alternative, particularly for complex fracture patterns [11].

Current concepts in the management of distal tibial fractures emphasize the importance of stable fixation while minimizing further damage to the surrounding soft tissues. ORIF with LCP and IMN is commonly used. The IMN and LCP have distinct biomechanical profiles. The IMN provides robust load sharing properties, making it suitable for fractures in which early mobilization is critical [15]. However, it can be less effective for managing rotational forces and maintaining alignment in comminuted fractures. In contrast, the LCP offers superior control over rotational stability but at the cost of increased soft tissue disruption [16]. Considering the pros and cons of IMN and LCPs, NPC has emerged as a promising technique that combines the benefits of both methods, offering enhanced stability and better alignment in complex fractures [10, 11].

Mechanically, the NPC technique offers several advantages over traditional IMN and plating methods. NPC combines the axial stability provided by intramedullary nails with the rotational stability of plates, which allows for more effective management of complex fracture patterns, thereby reducing the risk of nonunion and malalignment. Biomechanically, NPC has been shown to provide superior axial and torsional stability, compared with single-modality treatment. Intramedullary nailing alone tends to restrict axial movements but may cause higher shear movements, which can lead to complications, such as malunion [17]. The addition of a plate helps counteract these forces, thereby improving the overall stability and facilitating earlier weight-bearing [18].

In the current study, we investigated the outcomes of eight patients with complicated distal tibial fractures who were treated using the NPC technique. The findings demonstrated that NPC achieved good functional and radiographic outcomes, with an average OMAS score of 77.63 and an average AOFAS score of 82 at the 12-month follow-up. The average time to radiographic union and weight bearing was 7.8 and 3.4 months, respectively. These findings suggest that NPC is an effective treatment modality for managing complex distal tibial fractures. Therefore, we advocate for the application of the NPC method in specific cases, such as combined AO/OTA 42 and 43-C tibial fractures, distal tibial fracture nonunion, and open fractures with bone defects.

We distributed a web-based questionnaire to evaluate the acceptance and perception of NPC among trauma orthopedic surgeons. The survey included pre- and postoperative radiographs and clinical details of patients treated with NPC. Initially, many surgeons expressed a preference for traditional fixation methods, such as IMN or ORIF with locking plates. However, after reviewing the detailed cases and surgical techniques of the NPC, nearly all respondents indicated a willingness to consider NPC as a fixation method for similar fractures in the future.

This case series not only demonstrates the successful outcomes of ORIF with NPC but also highlights the potential for broader acceptance of this technique among orthopedic trauma surgeons. The distribution of a detailed questionnaire revealed that after reviewing the surgical methods and positive results associated with NPC, a significant number of surgeons expressed a willingness to consider NPC as a treatment option for complex distal tibial fractures. This shift suggests that increased awareness and understanding of the benefits of the NPC method could lead to its wider adoption in clinical practice.

Despite the promising results, this study has some limitations. First, the small sample size of only eight patients might not have provided a comprehensive representation of the broader population with distal tibial fractures. Second, the study lacks a control group that was treated with traditional methods, making it difficult to directly compare the effectiveness of NPC to other techniques. Third, a follow-up period of 12 months may be insufficient for assessing long-term outcomes and potential late complications. Finally, the retrospective study design may have introduced selection bias and affected the generalizability of the findings.

## Conclusion

The current study demonstrated that the NPC provides effective functional and radiographic outcomes for managing complicated distal tibial fractures. The results demonstrate that the NPC can achieve good union rates and functional scores with minimal complications. Positive feedback from the web-based questionnaire indicated a growing acceptance of the NPC among orthopedic trauma surgeons, suggesting that this technique could be widely adopted in clinical practice. Therefore, we advocate for the use of the NPC in complicated distal tibial fractures, including combined AO/OTA 42 and 43 fractures, nonunion of distal tibial fractures, and open fractures with bone defects.

## Supplementary Information

Below is the link to the electronic supplementary material.

Supplementary file1 (DOCX 2175 KB)

## Data Availability

The datasets used and/or analysed during the current study available from the corresponding author on reasonable request.
