# Supplementary material for: Evaluation of nail-plate construct fixation for complicated distal tibial fractures: a retrospective analysis of clinical and radiographic outcomes
Source: Eur J Orthop Surg Traumatol. 2026 Apr 15;36(1):173. doi: 10.1007/s00590-026-04704-2 (PMC13083477; doi:10.1007/s00590-026-04704-2)
Supplement: Supplementary file 1 — Supplementary file1 (DOCX 2175 KB) [file 590_2026_4704_MOESM1_ESM.docx]

**Supplementary Material: Web-based Questionnaire on the Use of Nail-Plate Combination for Complicated Distal Tibial Fractures**

The treatment choice for complex distal tibia fracture

Hello to all trauma orthopedic and orthopedic surgeons, we would like to seek your expert opinions on surgical methods through three simple cases. Thank you for your responses!

1。 Your current age? *

single choice

20-30

30-40

40-50

50-60

over 60

2。 You are currently at the clinical position of? *

single choice

Resident

Chief Resident/ Fellow

V1-4

V5-8

V9-15

over V16

3。 What is your current place of practice? *

single choice。

Medical center Regional hospital Local hospital Clinic

4。 What is your monthly volume of trauma surgeries? *

single choice。

> 30 cases

20-30 cases

10-20 cases

< 10 cases

Case 1 summary

This is a 39-year-old female with no chronic diseases and a history of cesarean section at the age of 25.

She was hit by a car while riding a motorcycle, with no head trauma or neurological symptoms, and no pain in the chest or abdomen. However, she experienced deformity and pain in the right lower limb, with no open wound.

The medical team opted for external fixation as the first stage of treatment.

AP view


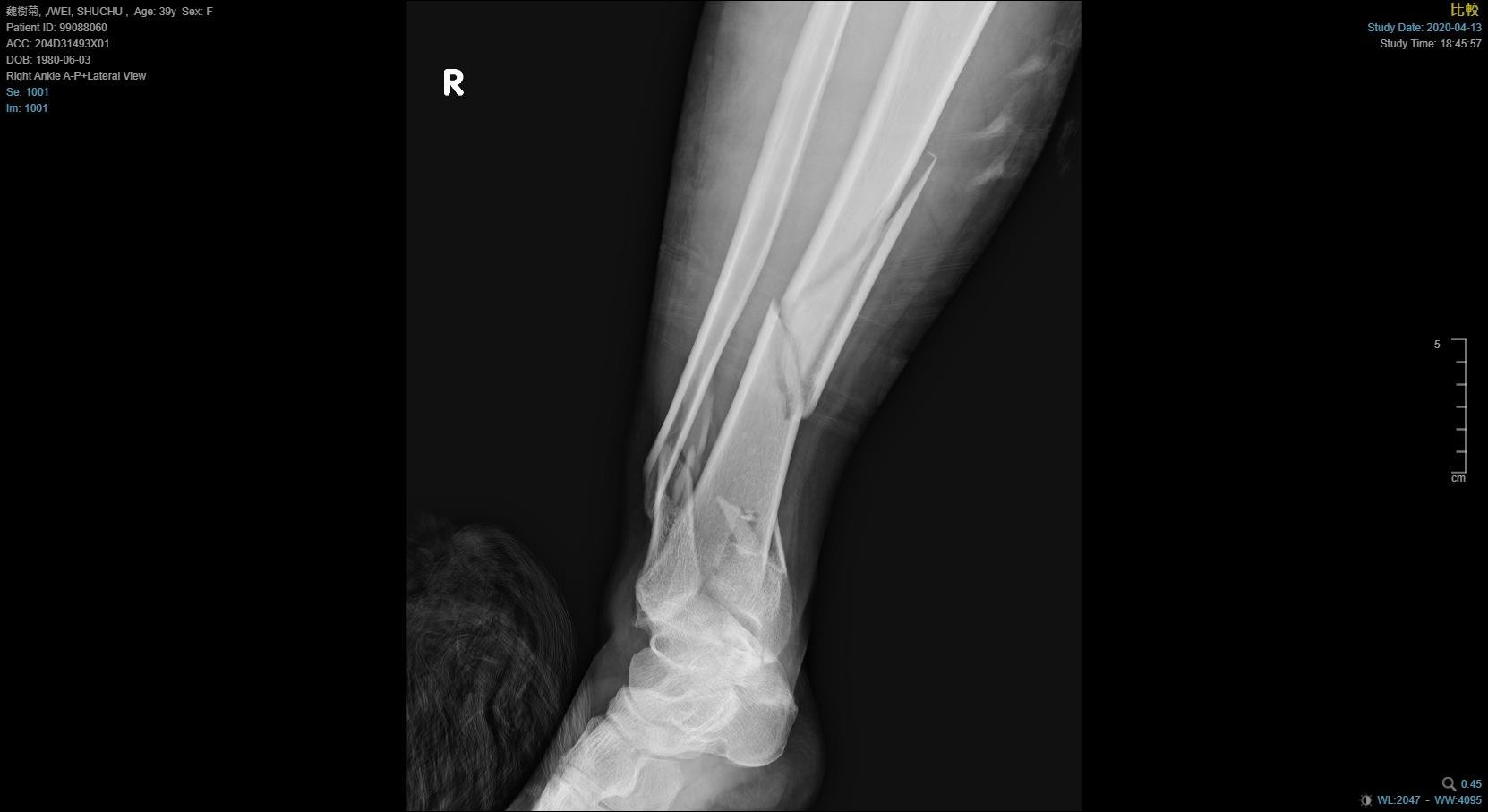


Lateral view


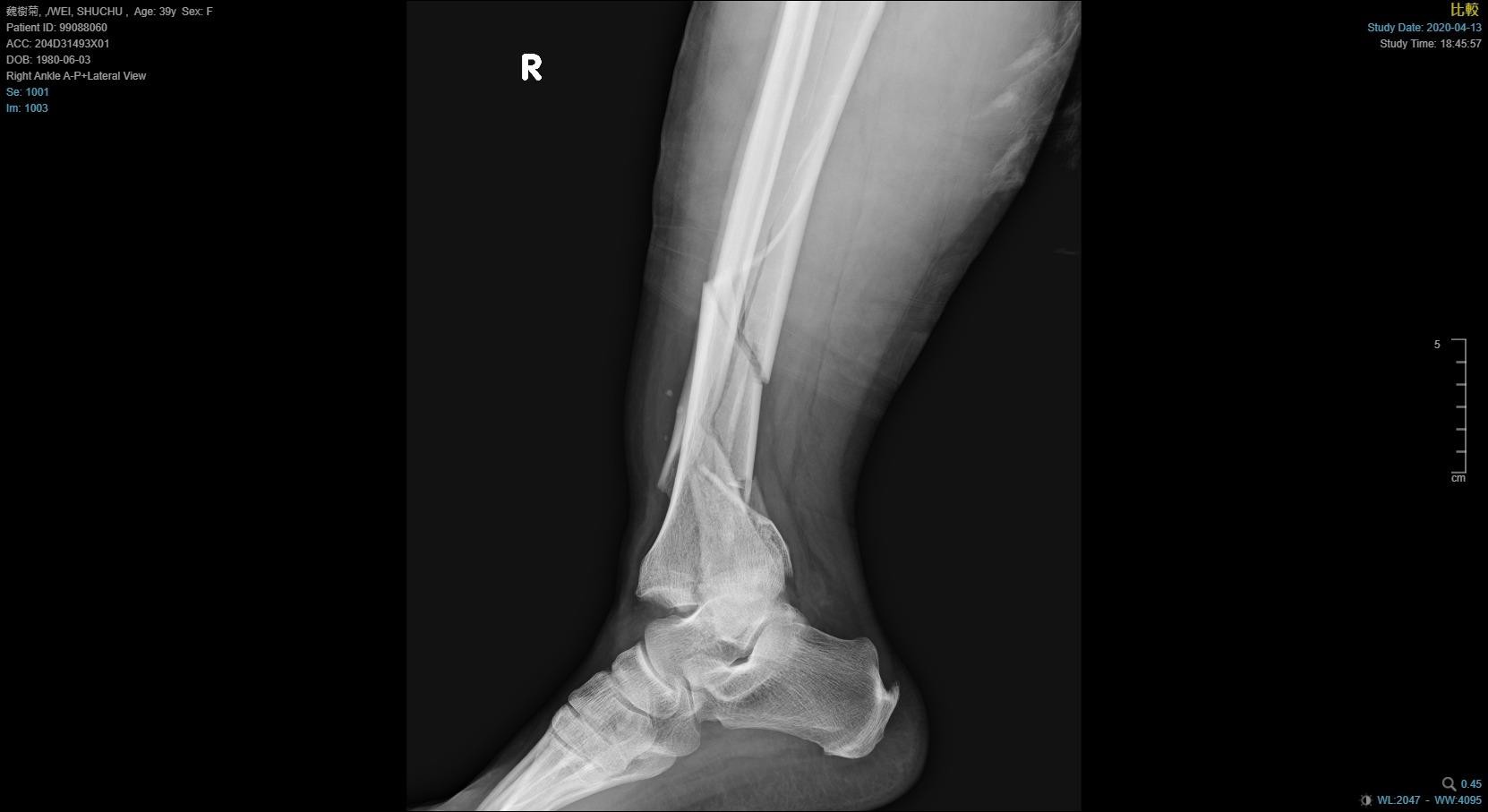


External ﬁxation


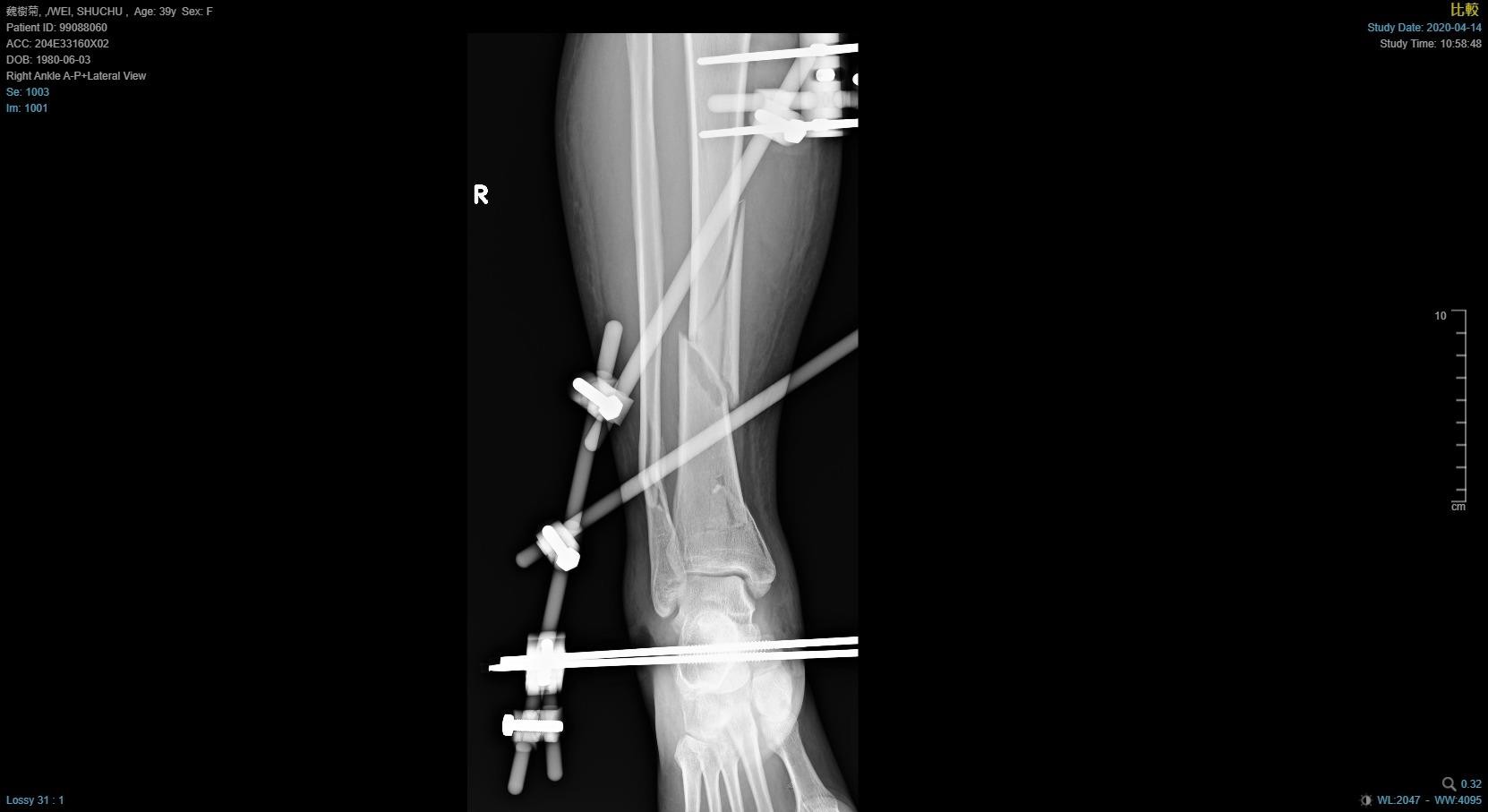


3D CT video

[Video link](https://www.youtube.com/watch?v=SMyJPyDEI5Y)

5。 For the second stage of internal ﬁxation, what surgical method would you * "prioritize" as your ﬁrst choice? (If choosing "other," please describe your reconstruction approach.)

single choice。

ORIF with single locking plate (LCP)

ORIF with both anterolateral and medial LCP plates ORIF with intramedullary nailing

Definite external fixation

others：

Case 2 summary

This is a 70-year-old male with chronic conditions including type 2 diabetes and hyperlipidemia. He has a surgical history of urolithiasis extraction at the age of 58.

He was hit by a car while walking, sustained a head injury with loss of consciousness at the scene, but was conscious upon arrival at the emergency room. However, CT scans revealed intracerebral hemorrhage (ICH) and subdural hematoma (SDH), with no pain in the chest or abdomen. The left lower limb was deformed with an open wound (Gustilo type II). The neurosurgery consultation suggested that orthopedic fixation could be performed first, followed by observation and treatment in the neurosurgical ICU.

The medical team opted for external fixation as the first stage of treatment.

For the definitive fixation, a single L plate was initially used for treatment, but subsequently, there was a complication of plate implant failure.

Appearance


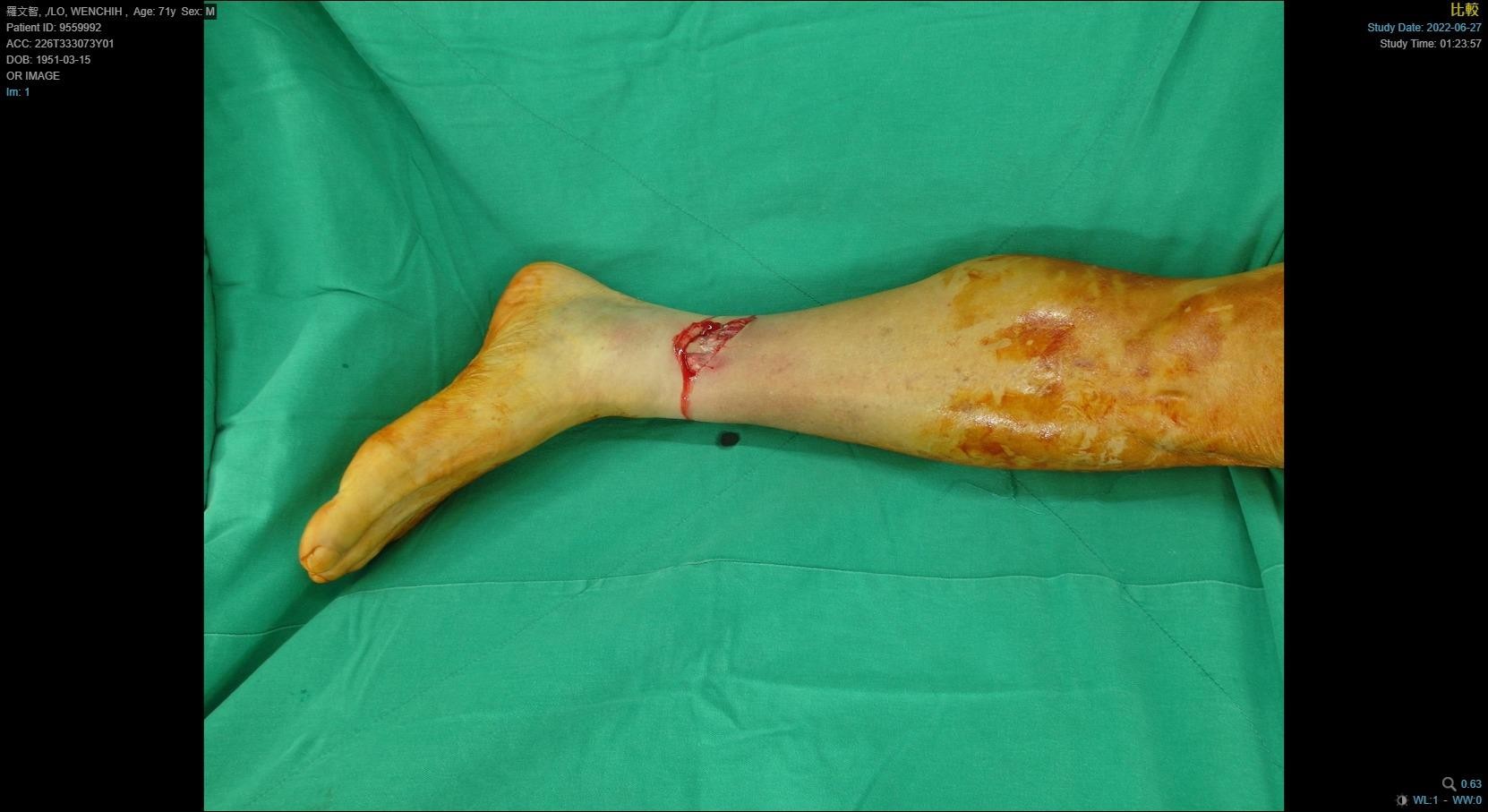


AP view


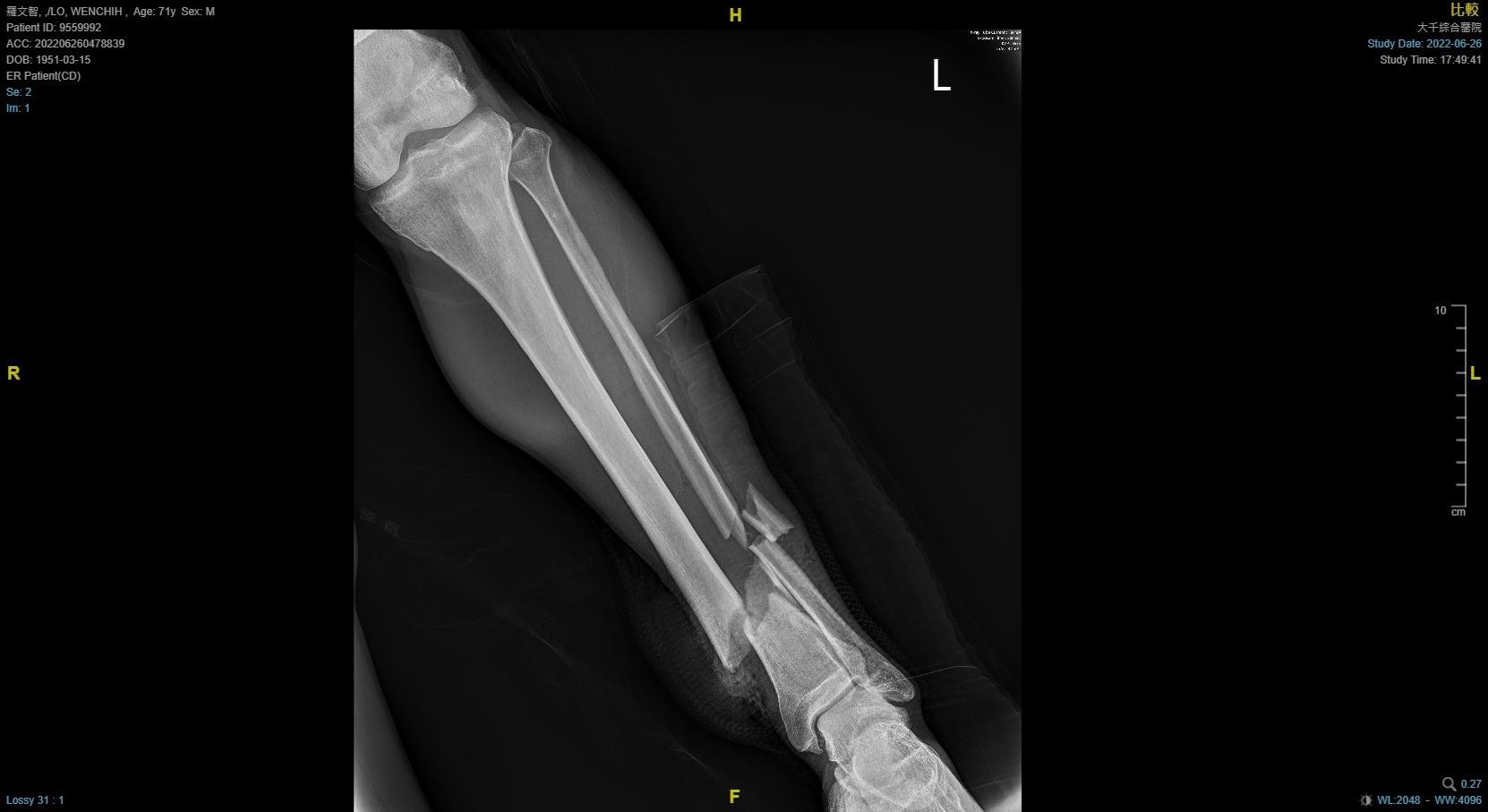


Lateral view


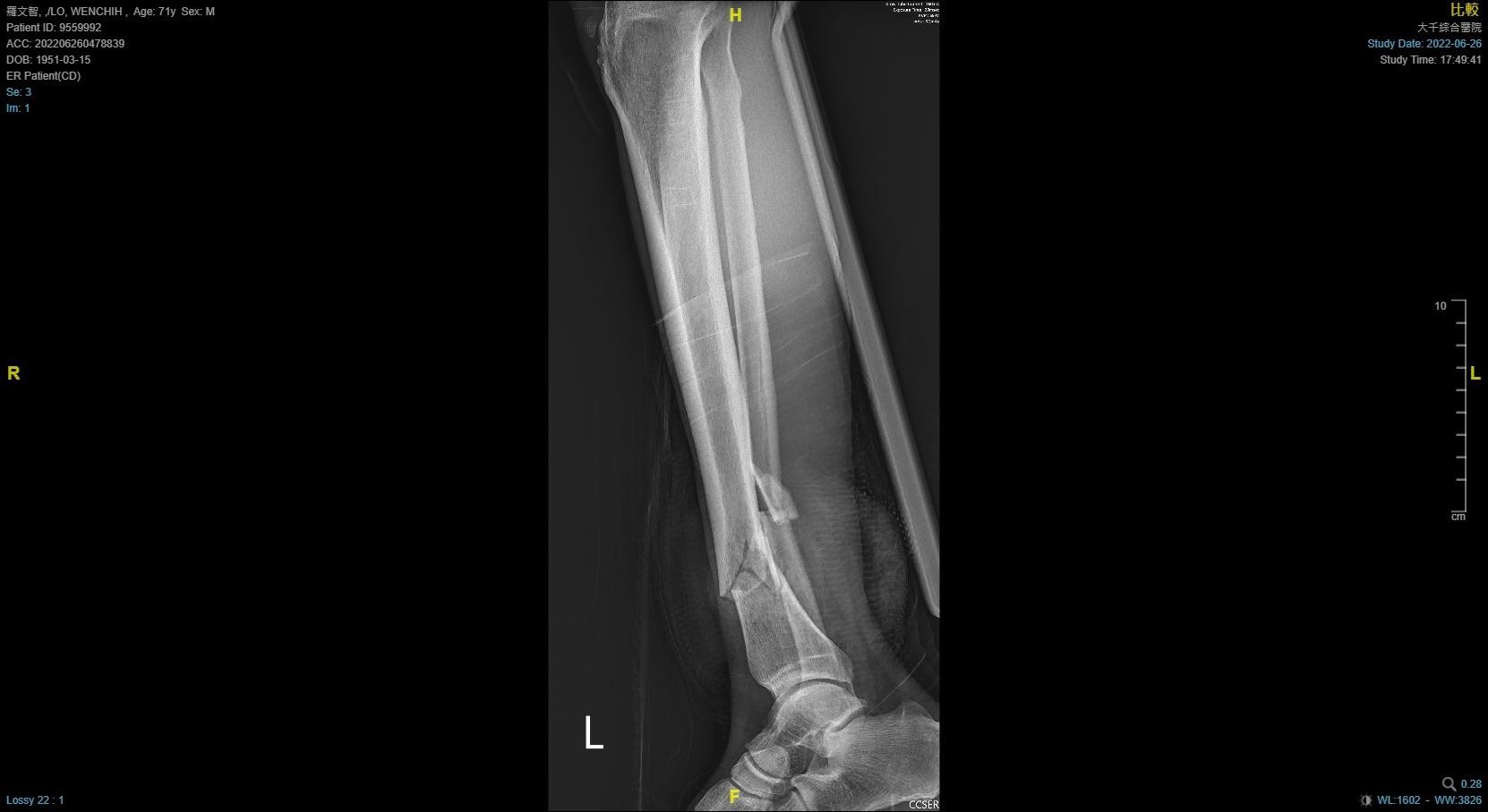


External ﬁxation


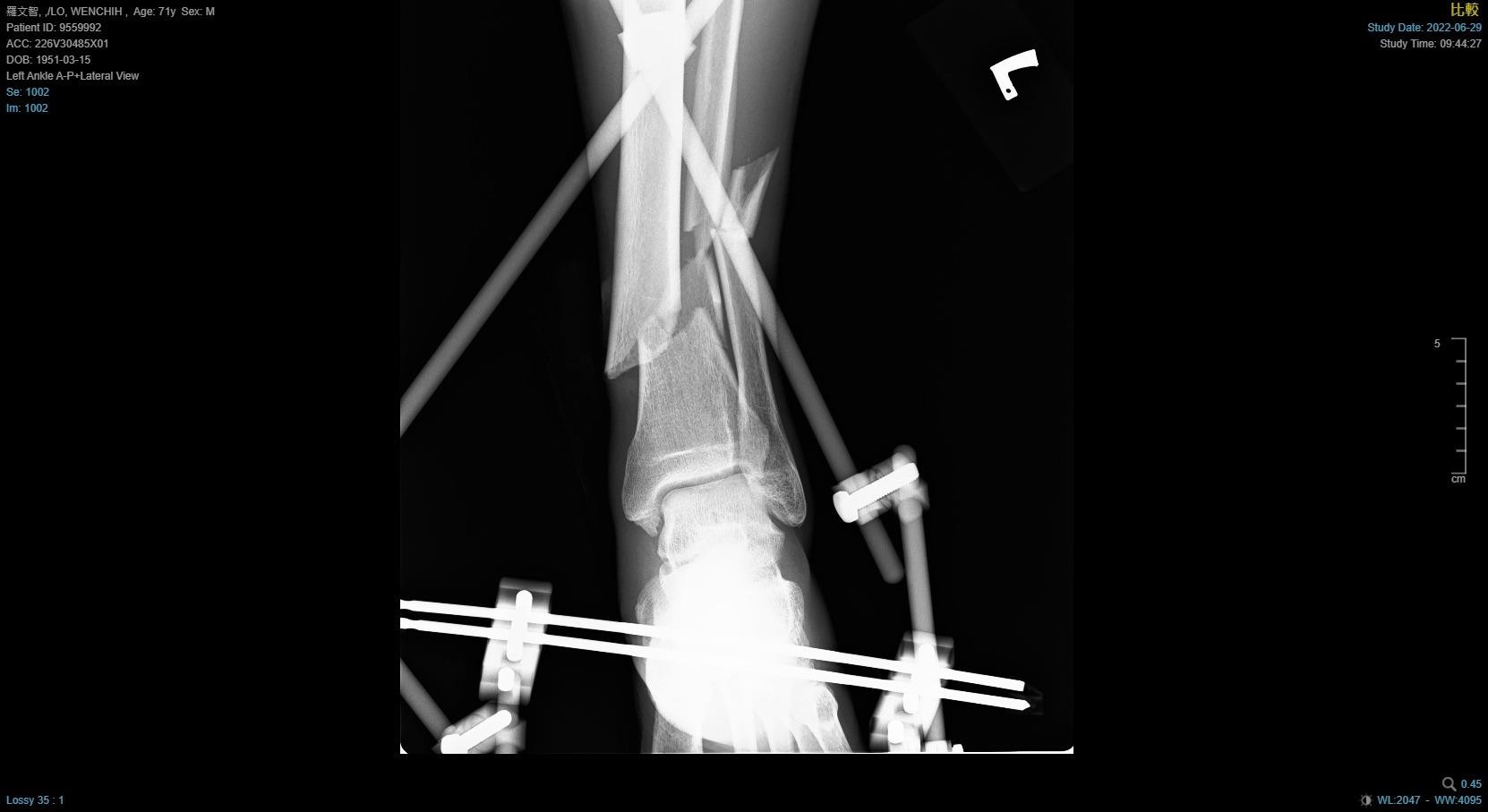


External ﬁxation


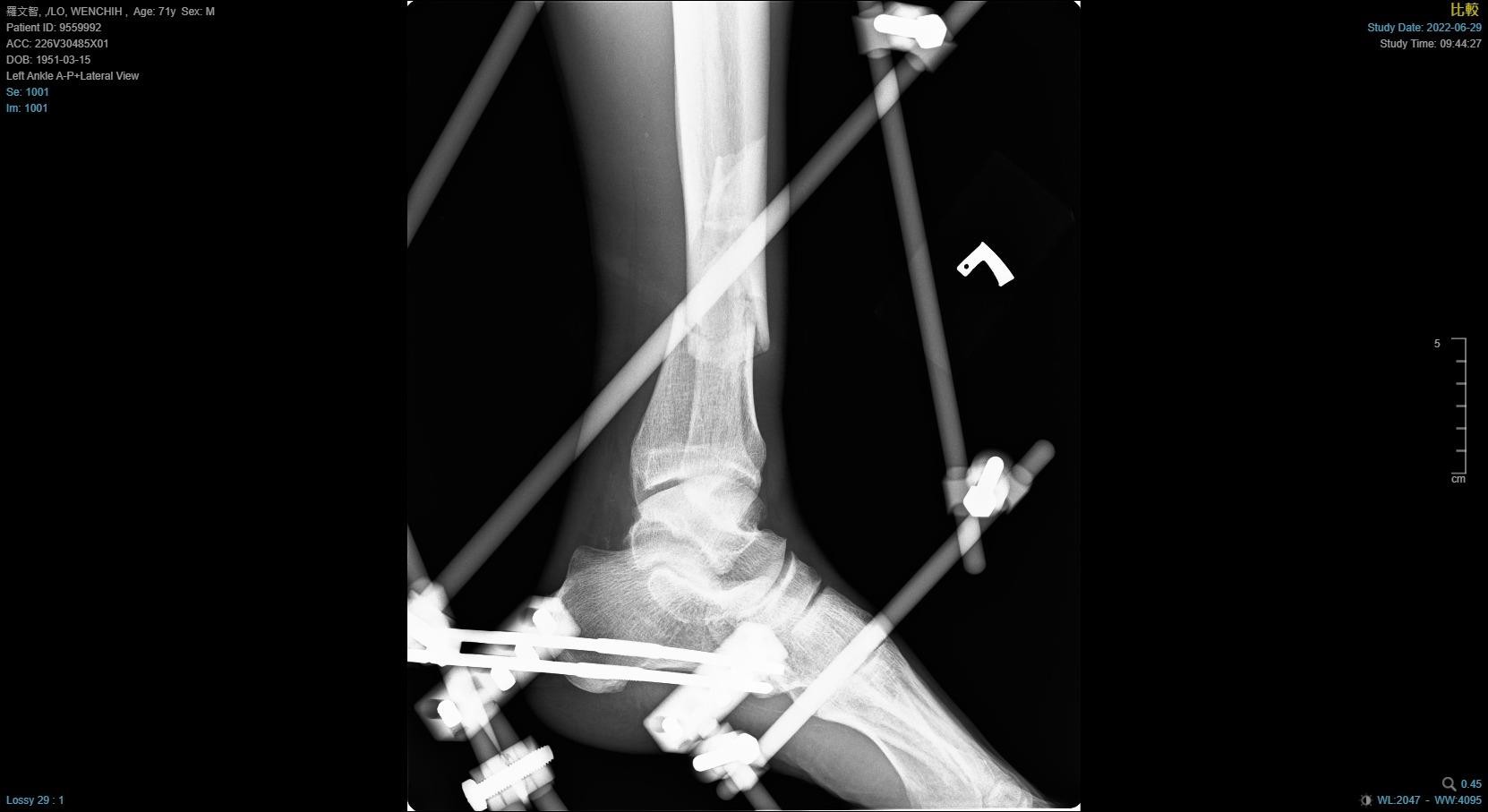


ORIF with plate ﬁxation


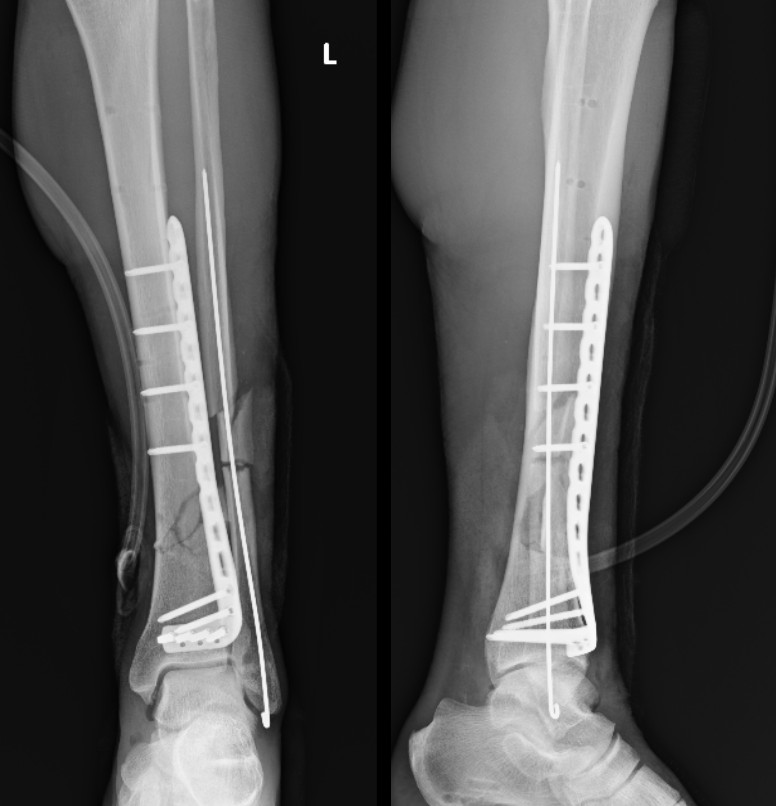


Plate implant failure after 4m


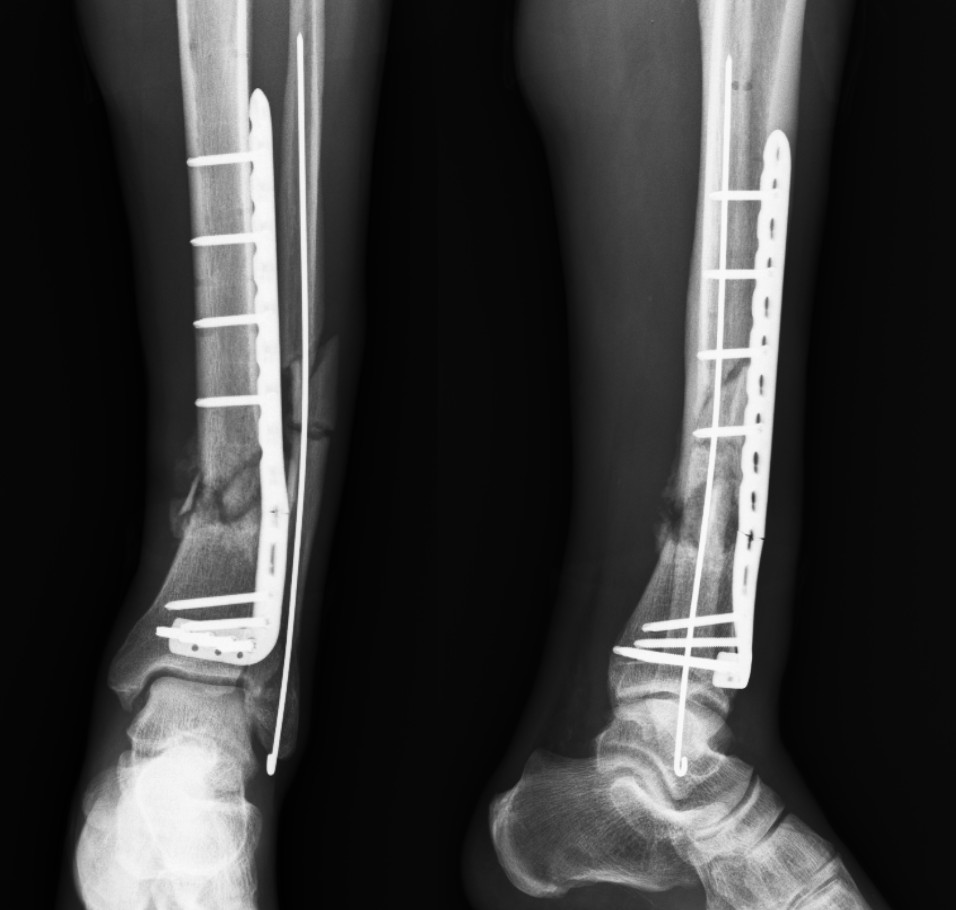


6。 For such a complication, what surgical method would you "prioritize" as your *

ﬁrst choice? (If choosing "other," please describe your reconstruction approach.)

single choice。

Revision ORIF with single locking plate (LCP)

Revision ORIF with both anterolateral and medial LCP plates Revision ORIF with intramedullary nailing

others：

Case 3 summary

This is a 57-year-old male with a chronic condition of gout and no past surgical history.

He was injured in the left lower limb by a steel rod during work, resulting in deformation and an open wound (Gustilo type IIIA).

The medical team opted for external fixation as the first stage of treatment.

Appearance


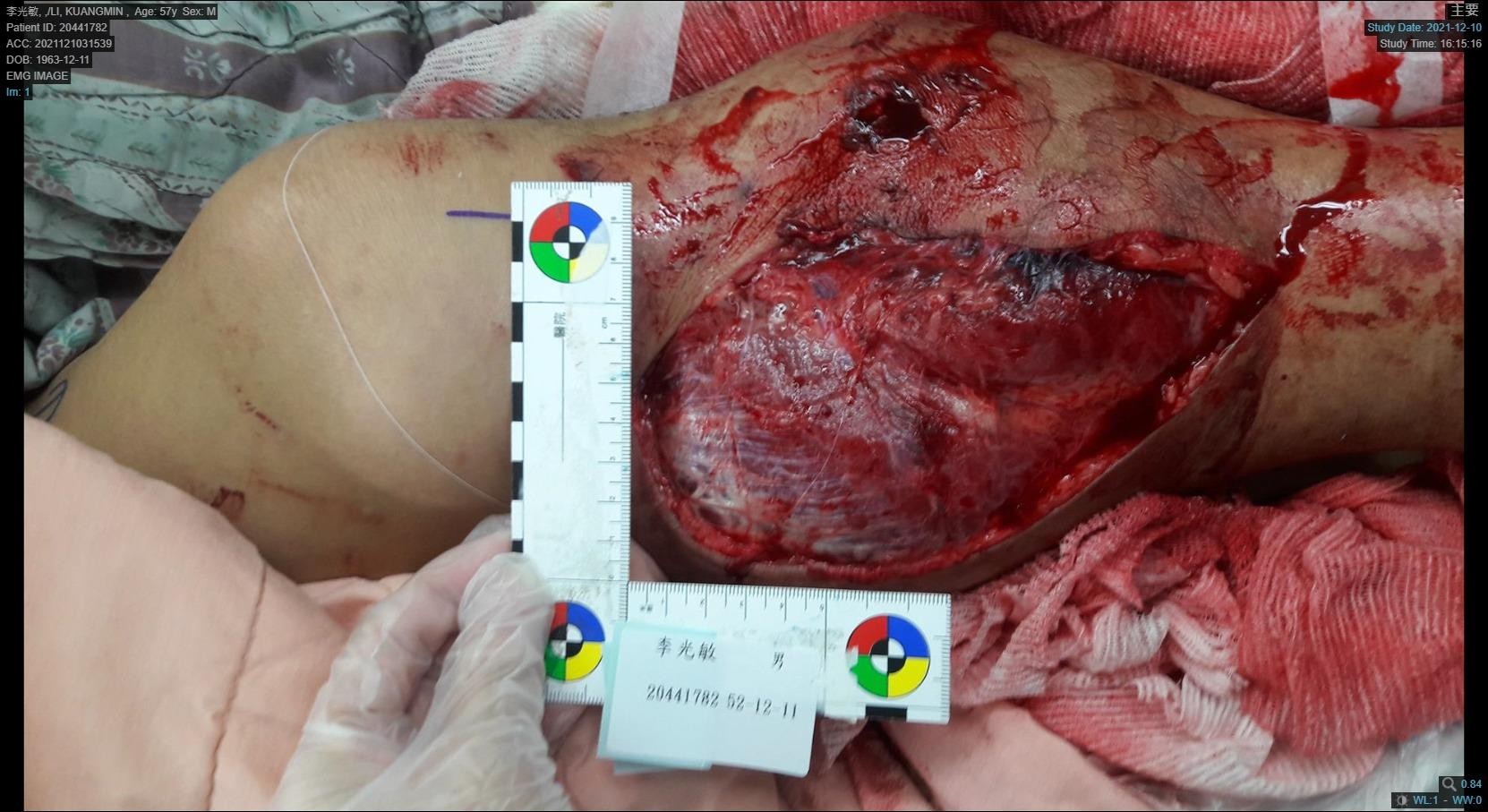


AP and Lateral view


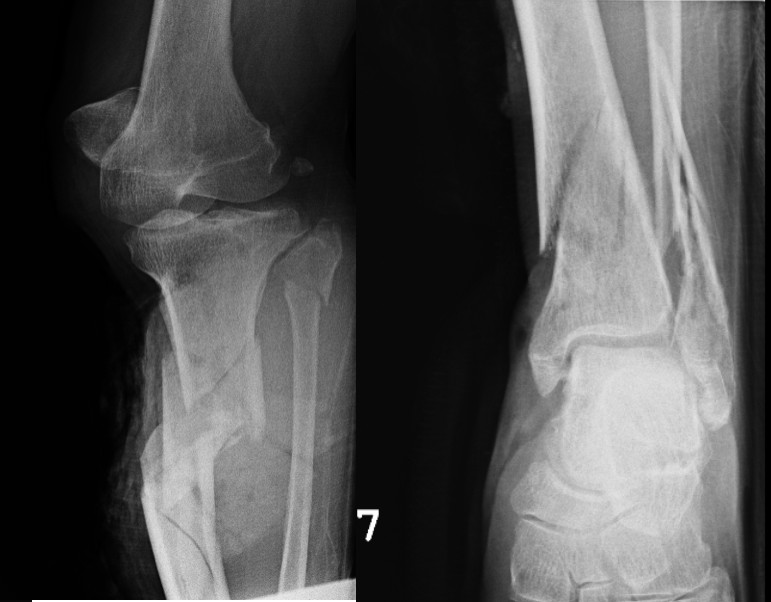


External ﬁxation


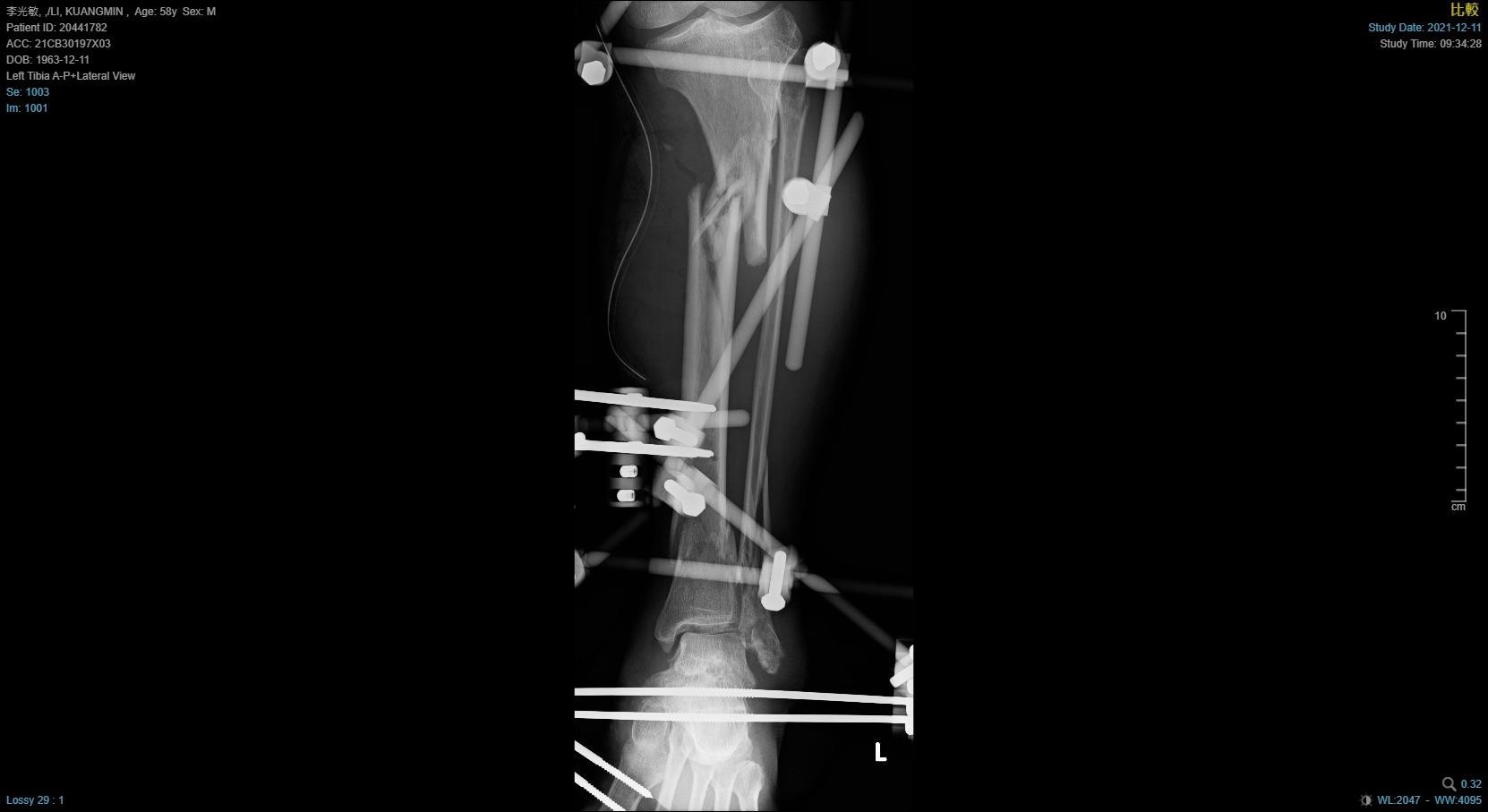


External ﬁxation


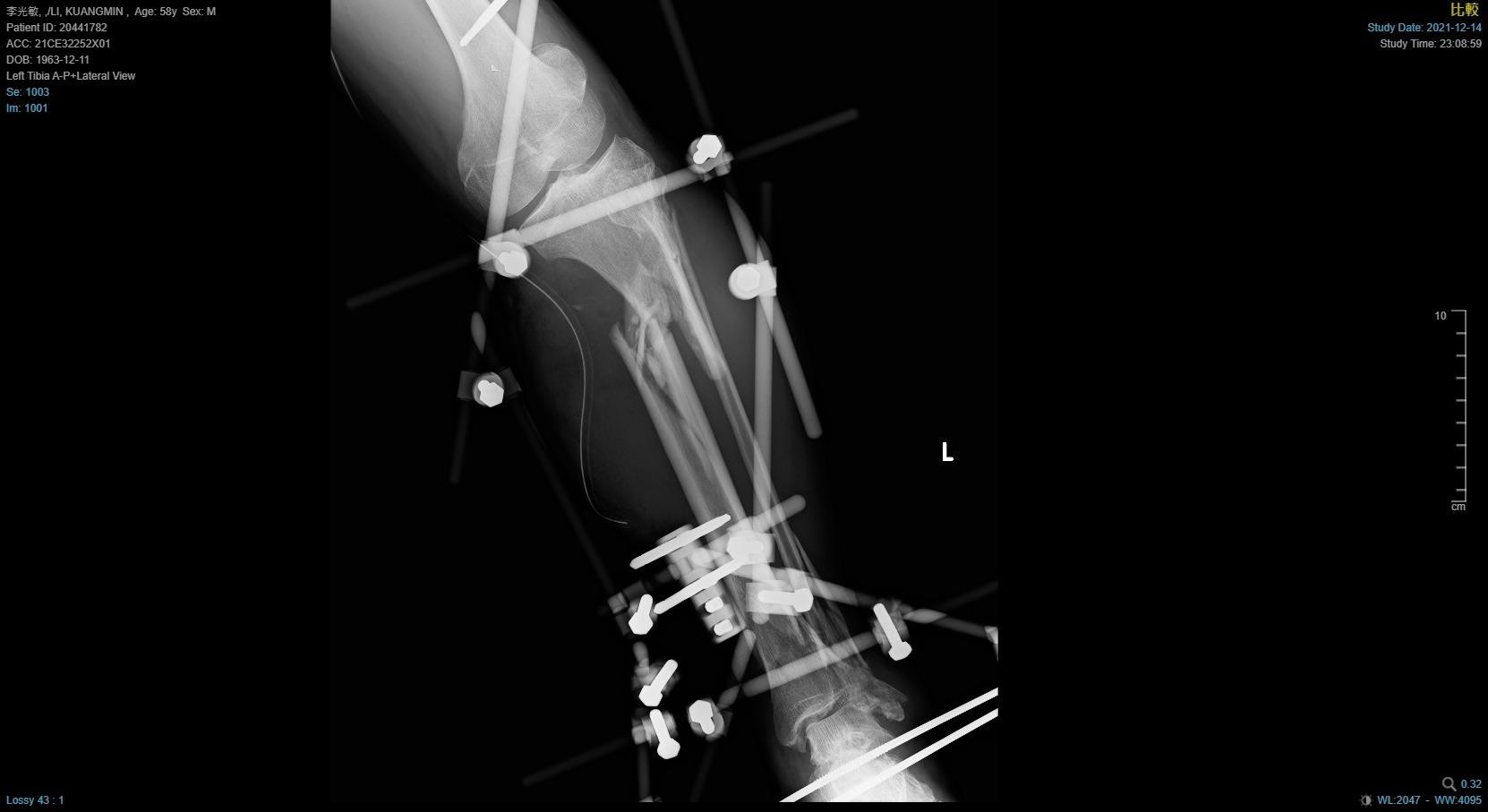


3D CT

[Video link](https://youtu.be/_iYUsQg05kM)

7。 For the second stage of internal ﬁxation, what surgical method would you * "prioritize" as your ﬁrst choice? (If choosing "other," please describe your reconstruction approach.)

single choice。

ORIF with single locking plate (LCP)

ORIF with both anterolateral and medial LCP plates ORIF with intramedullary nailing

Definite external fixation

others：

The application of Nail Plate Combination (NPC) in distal tibia fractures

[Reference](https://pubmed.ncbi.nlm.nih.gov/27768628/)

The application of the Nail Plate Combination (NPC) is primarily for the proximal tibia, but it can also be utilized in the distal tibia to counteract the instability that leads to implant failure or nonunion.

Although the use of NPC for distal tibia fractures may not be common, it serves as a useful and reliable method in managing fractures at the very distal end of the tibia. The combination of an intramedullary nail and plate not only aids in achieving and maintaining fracture reduction before the placement of the nail but more importantly, it provides a stable construct. This stability allows for immediate weight-bearing and resists the pendulum effect that could lead to implant failure.

Post OP, case 1


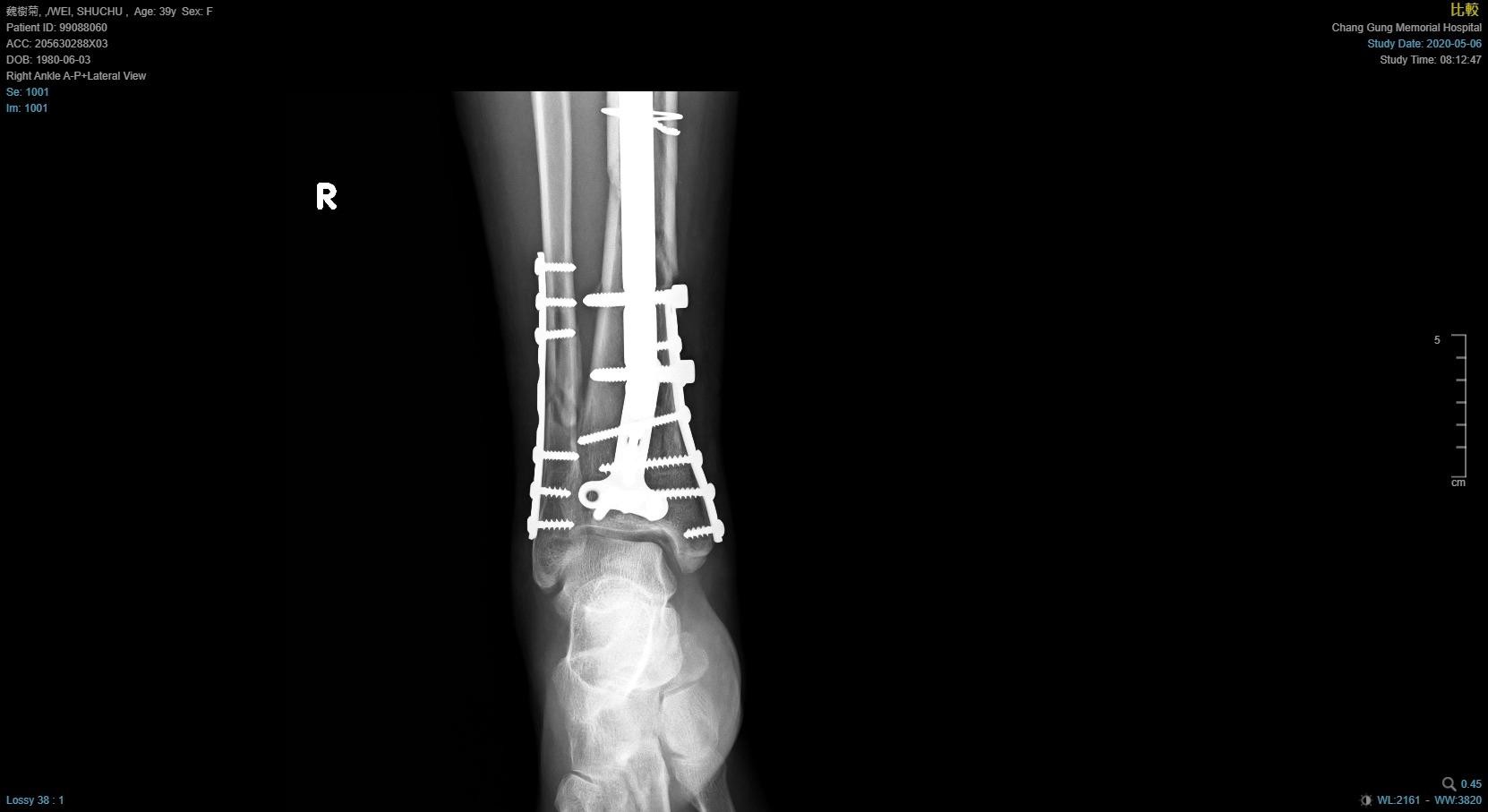


Revision, case 2


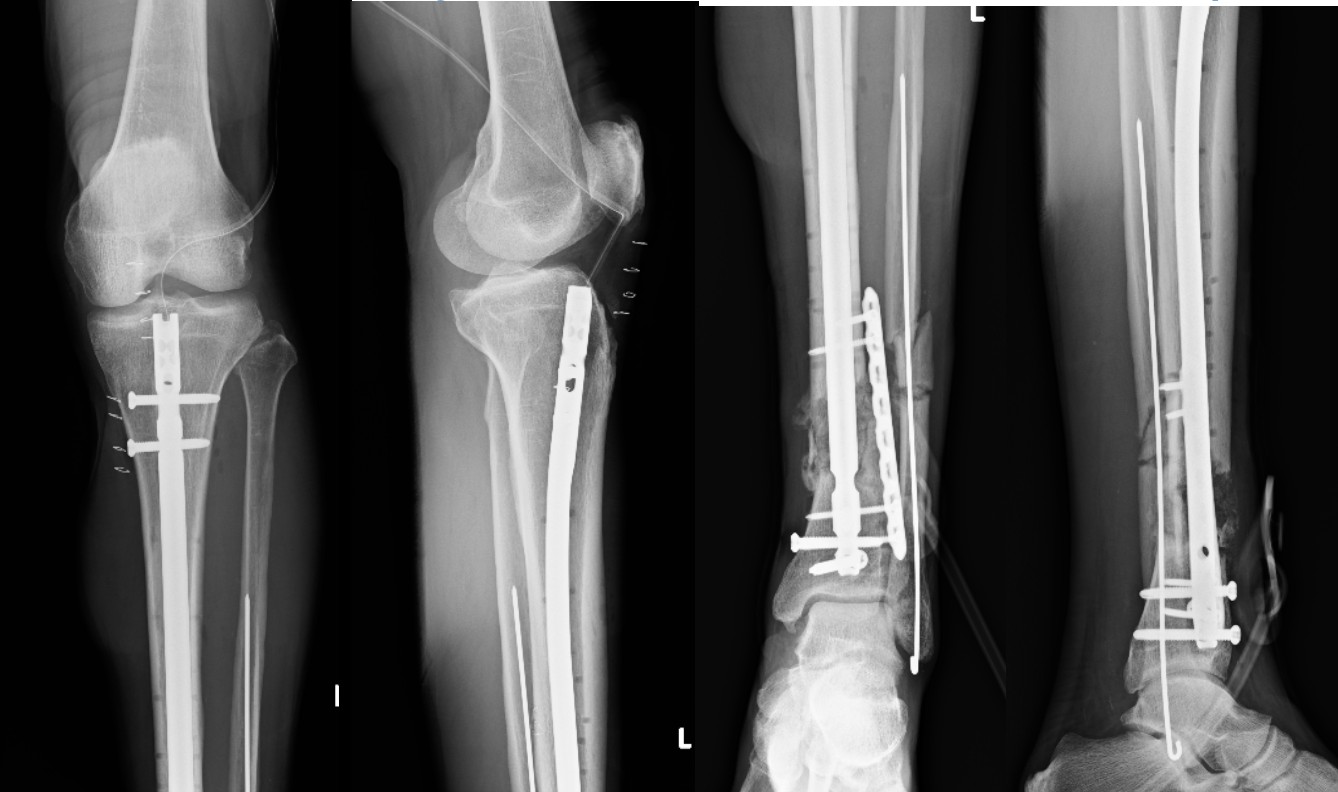


Post revision op 9m, case 2


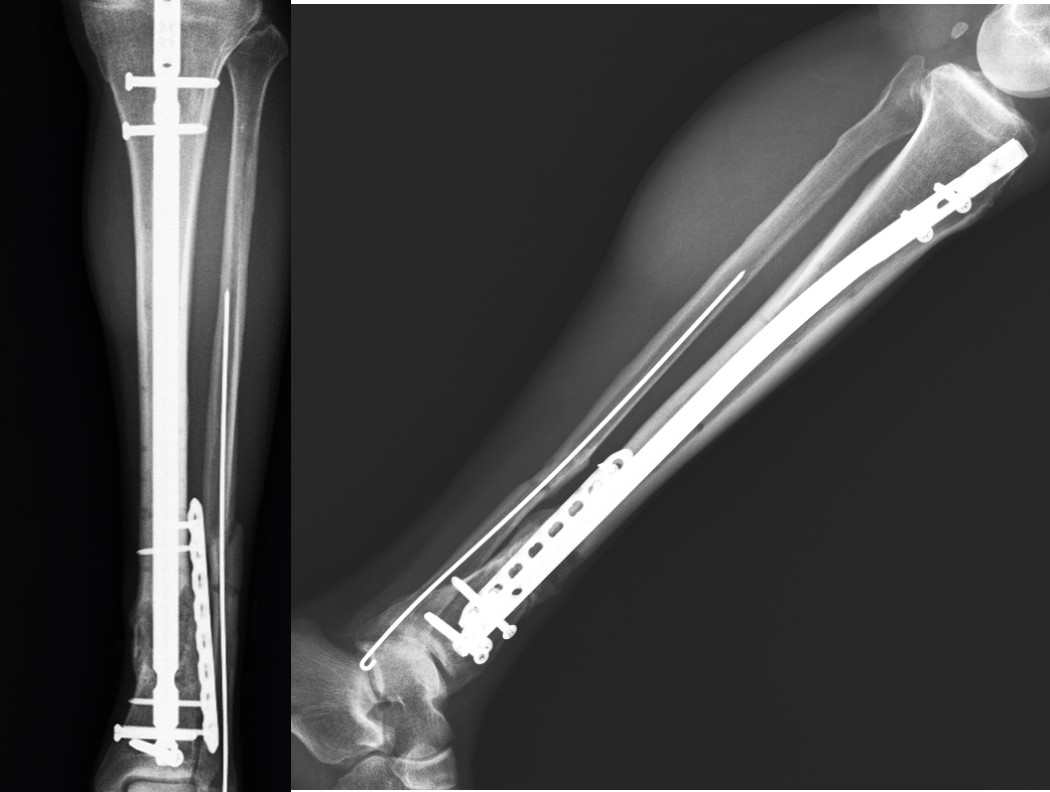


Post OP case 3


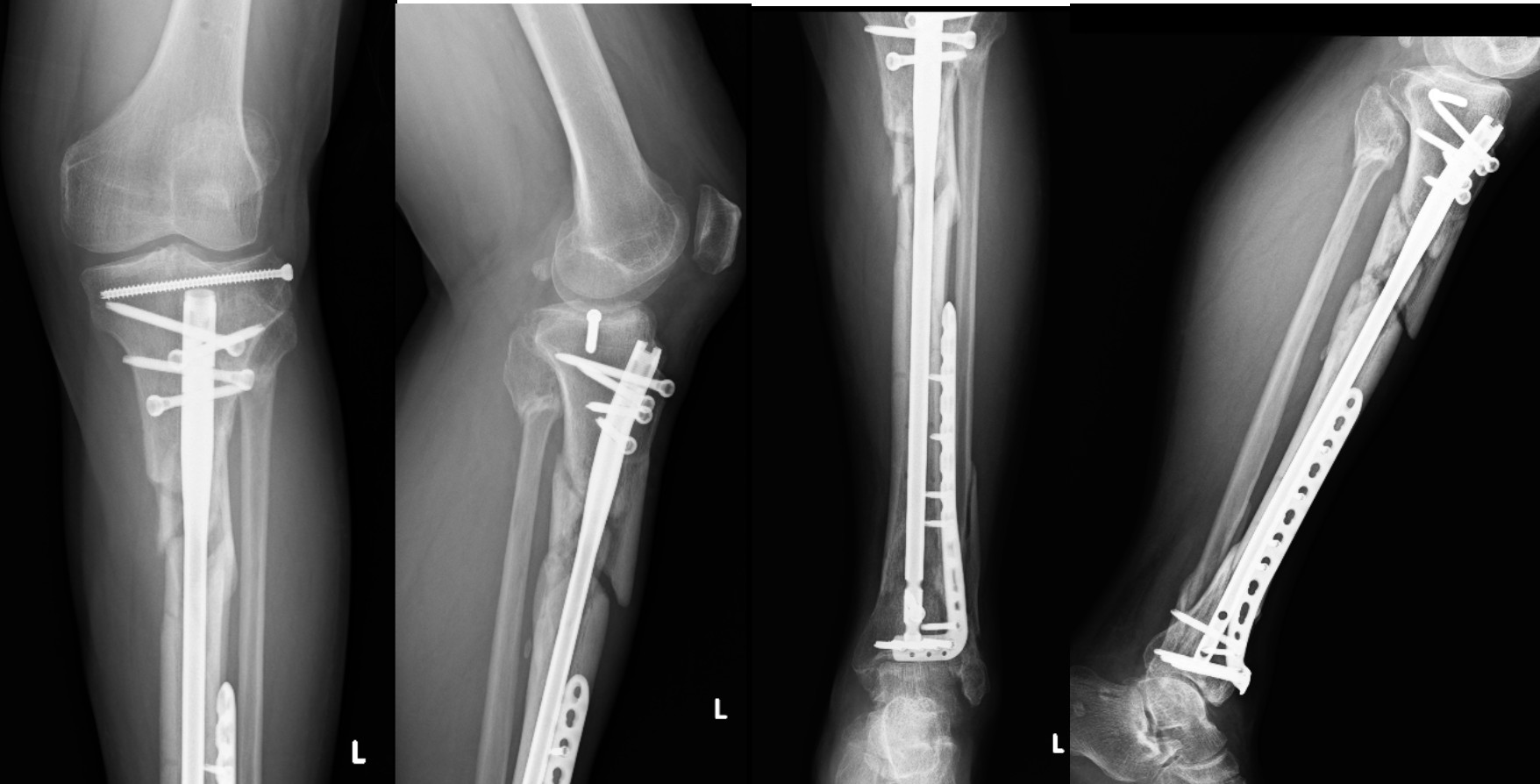


Post OP 10 month Case 3


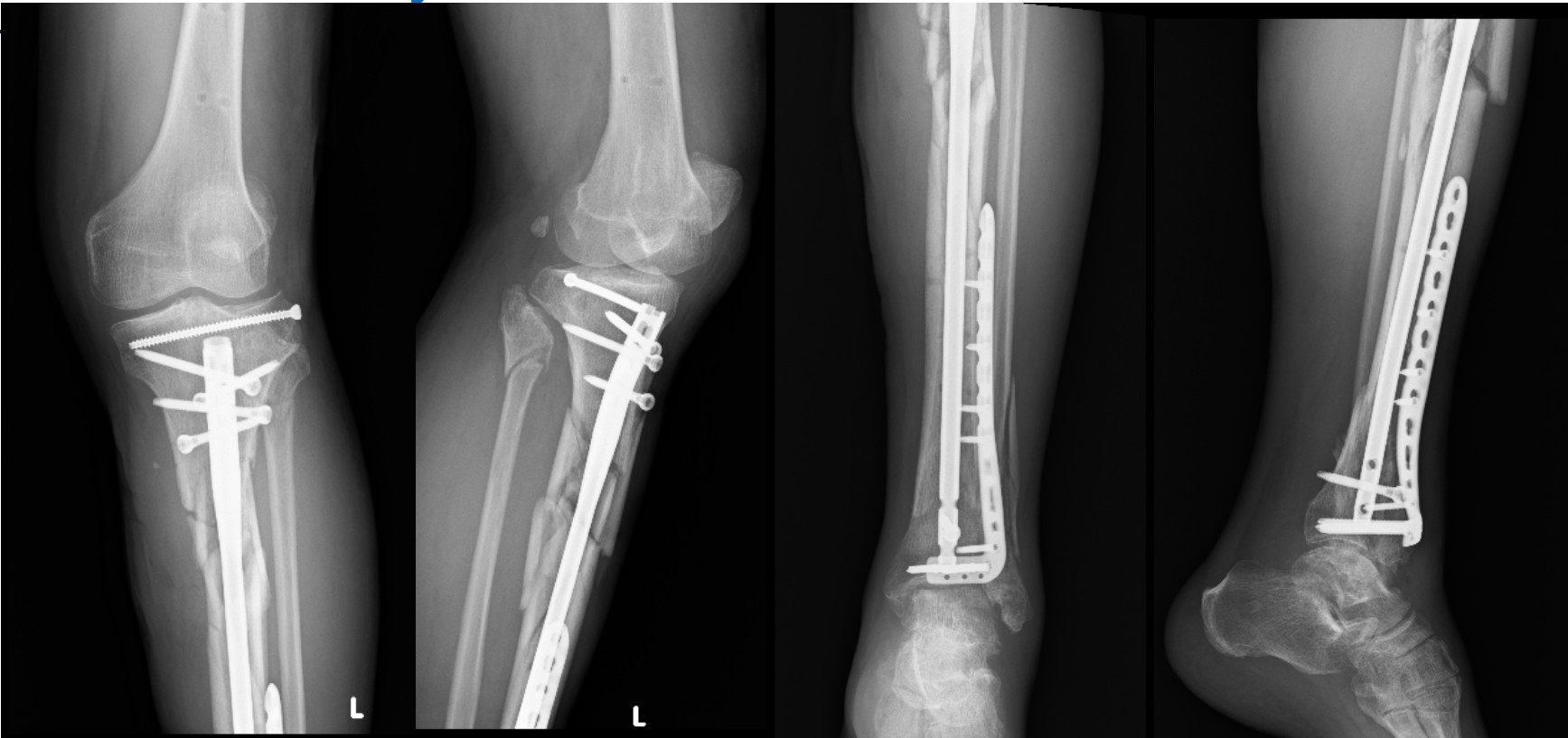


8。 *After learning about the clinical outcomes and relevant paper summaries of NPC applications in the distal tibia, would you reconsider using NPC to treat these three cases?

single choice。

Yes

No

9。 If you chose "No", please point out the reasons:

Thank you for completing the questionnaire
